# Genome-wide identification and expression analyses of R2R3-MYB transcription factor genes from two Orchid species

**DOI:** 10.7717/peerj.9781

**Published:** 2020-09-01

**Authors:** Honghong Fan, Manli Cui, Ninghong Li, Xujuan Li, Yuxuan Liang, Lin Liu, Yongping Cai, Yi Lin

**Affiliations:** 1 School of Life Sciences, Anhui Agricultural University, Hefei, China; 2 Faculty of Forestry, University of British Columbia, Vancouver, Canada

**Keywords:** *Dendrobium officinale*, *Phalaenopsis aphrodite*, Orchidaceae, R2R3-MYB, Gene family

## Abstract

MYB transcription factors play important roles in different plant biological processes during plant growth, development and stress response. In this study, 101 (*DoMYB1-101*) and 99 (*PaMYB1-99*) *R2R3-MYB* genes were identified in the genomes of *Dendrobium officinale* and *Phalaenopsis aphrodite*, respectively. To classify the isolated candidate genes, the *R2R3-MYB* genes from *A. thaliana* were selected as references. As a result, all identified *DoMYB* and *PaMYB* genes were classified into 22 subfamilies. Phylogenetic analysis revealed that S21 had the largest number of members of all the subfamilies. The numbers of introns, exons and conserved sequences in all of the identified genes are different. In addition, 20* DoMYB* genes from six subfamilies were selected for further analysis of tissue-specific expression and responses to various abiotic stresses treatments. The results showed that all of the *DoMYB* genes in S4 and S19 subfamilies exhibited the highest relative expression levels in flowers. And five *DoMYB* genes including *DoMYB31*, *DoMYB40*, DoMYB49, *DoMYB52* and *DoMYB54*, responded to the stress response. These results may provide useful information for further studies of the R2R3-MYB gene family.

## Introduction

As a superfamily of transcription factors (TFs), MYB proteins comprise one of largest gene families in plants. They play important roles in controlling metabolism regulation and abiotic or biotic responses during the whole processes of plant growth and development (Chen et al., 2005; Du et al., 2012; Dubos et al., 2010; Peng et al., 2016; Nesi et al., 2001). For example, MYB TFs are involved in the regulation of various phytochemical biosynthesis pathways (Adato et al., 2009). They are closely related to the regulation of several developmental processes, secondary cell wall biosynthesis, the development of stem apical meristems, and the synthesis of plant lignin (Zhong et al., 2008; Kirik et al., 1998).

The MYB gene was firstly identified in maize and then successively isolated from many other plants. MYB TFs contained a highly conserved DNA binding domain (R) with 50 to 53 amino acids and a conserved tryptophan at every 18 amino acids. Based on the number of special R domains, MYB TFs can be divided into four categories, namely, R1R2R3-MYB, R2R3-MYB, R1-MYB, and 4R-MYB (Dubos et al., 2010). Among them, R2R3-MYB contains two conserved domains (2R) with five tryptophan residues in the repeat sequence, which forms a helix-turn-helix motif at the N-terminus and plays a vital role in maintaining the stability of the MYB TFs (Rabinowicz et al., 1999; Stracke, Werber & Weisshaar, 2001).

Recently, the *R2R3-MYB* gene family has been identified in many plants. There are at least 126, 109, 157 and 192 members of *R2R3-MYB* genes in *Arabidopsis thaliana*, *Oryza sativa*, *Zea mays* and *Populus trichocarpa*, respectively (Dubos et al., 2010; Du et al., 2012; Katiyar et al., 2012; Wilkins et al., 2009). Clustering analysis of the *R2R3-MYB* gene family in *A. thaliana* revealed that the *R2R3-MYB* genes could be divided into 25 subfamilies (Matus, Aquea & Arce-Johnson, 2009; Stracke, Werber & Weisshaar, 2001). Of all these subfamilies, the S4 subfamily was involved in the synthesis of various alkenes in *Oil palm* (Li et al., 2017a; Li et al., 2017b). The S6 subfamily was proved to be closely related to the synthesis of carotenoids and anthocyanins (Li et al., 2017a; Li et al., 2017b). The S7 subfamily was demonstrated to play a regulatory role in the terpenoid synthesis pathway in *A. thaliana* and *M. spicata* (Reddy et al., 2017; Stracke et al., 2007).

The orchid species are widely distributed in the world. For their ornamental value and medical use, four species of Orchidaceae including *Apostasia shenzhenica*, *Vanilla fragrans*, *Phalaenopsis aphrodite* and *Dendrobium officinale* have completed the whole genome sequencing (Zhang et al., 2017). Further biological evolution analysis revealed that orthologous genes might contribute to evolutionary innovations of nonfunctional, subfunctional or new functional genes after the evolutions of common ancestors for plants (Riechmann et al., 2000). MYB TFs are involved in all aspects of plant development and metabolism. Nevertheless, the reports on MYB TFs in orchids are few. In this study, the *R2R3-MYB* gene families were identified from *D. officinale* (*DoMYB*) and *P. aphrodite* (*PaMYB*). The analysis of the classification, phylogenetics, and chromosomal distribution was conducted. Otherwise, the expression patterns of the *DoMYB* genes were analyzed. The results may provide novel insights into the roles of MYB transcription factors in *D. officinale*, *P. aphrodite*, and other orchid plants.

## Materials & Methods

### Identification and classification of *R2R3-MYB* genes in *D. officinale* and *P. aphrodite*

The genomes of *D. officinale* (https://www.ncbi.nlm.nih.gov/bioproject/262478) and *P.aphrodite* (http://orchidstra2.abrc.sinica.edu.tw/orchidstra2/padownload.php) were downloaded to identify the candidate *R2R3-MYB* genes as previously described by Cao et al. (2016). Specifically, the Hidden Markov Model (HMM) in Pfam2 database was used to generate conserved domains (PF00249). It was then used to blast against the genome (*E*-value <1e^−3^) to obtain candidate *R2R3-MYB* genes. All candidate sequences were confirmed with the SMART tool (http://smart.embl-heidelberg.de). The uncertain *R2R3-MYB* proteins were filtered out. For further analysis of the phylogenetic relationships for the *R2R3-MYB* gene members in *D. officinale* and *P. aphrodite*, a phylogenetic dendrogram was constructed based on the full sequences of candidate proteins using MEGA5.2 software with the neighbor-joining (NJ) method. In detail, a phylogeny test was performed using the bootstrap method with 1000 replicates, substitution with the Possion model, and Gaps/Missing Data treatment with pairwise deletion. The uniform rates were used as the rates among sites. The number of threads is 7 (Tamura et al., 2011). The gap open and gap extend are -2.9 and 0, respectively. According to the classification of MYB subfamily in *A. thaliana*, the *R2R3-MYB* gene members were eventually divided into 25 subfamilies (Matus, Aquea & Arce-Johnson, 2009; Stracke, Werber & Weisshaar, 2001).

### Intron-exon structure and domain analysis of R2R3-MYB genes from *D. officinale* and *P. aphrodite*

The Gene Structure Display Server 2.04 (GSDS) was used to analyze the exons-introns structures of the obtained full sequences (Cao et al., 2016, Guo et al., 2007). The MEME suite (Multiple Expectation Maximization for Motif Elicitation, version 5.1.0) was employed to analyze the conserved motif (Bailey et al., 2006). The parameters were set as follows, 0 or 1 occurrence per sequence to be distributed in sequences; maximum number of motifs to find, 8; minimum width of motif, 6; maximum width of motif, 100; and the motif must be present in all members within the same subgroup. Subsequently, MAST XML output was used to analysis protein databases and finally redraw motif pattern. The annotations of the conservative patterns were performed using Pfam and SMART.

### Calculations of synonymous substitutions (Ks) and nonsynonymous substitutions (Ka)

According to the phylogenetic tree, two sequences of candidate genes with similar genetic relationships were isolated. The gene pairs were compared using DNAMAN software. Gene pairs with a consistency greater than 60% were selected for the calculation of Ka (non-synonymous substitution) and Ks (synonymous substitution). The values of *Ka*∕*Ks*, *Ka*, and *Ks* were calculated using DnaSP 5.0 software (Librado & Rozas, 2009). A *Ka*∕*Ks* ratio of 1.0 has been suggested to be a useful cut-off value to identify genes under positive selection. Generally, *Ka*∕*Ks* < 1.0 indicates purifying or negative selection. *Ka*∕*Ks* = 1.0 shows neutral selection, and *Ka*∕*Ks* > 1.0 means positive selection (Zhang et al., 2006).

### R domain and gene ontology (GO) annotation analysis

The sequences of the R2 and R3 domains of 101 *DoMYBs* and 99 *PaMYBs* were aligned using MEGA 5.0 to identify their features in the sequences. The multiple alignment files for these domains were submitted to WebLogo (http://weblogo.berkeley.edu/logo.cgi) using the default settings to acquire sequence logos as previously described by Cao et al. (2016). The protein sequences of *DoMYBs* and *PaMYBs* were aligned to the NCBI non-redundant protein database by BLASTp using Blast2GO software with the default parameters (Conesa et al., 2005). The functions of the R2R3-MYB proteins were predicted by GO annotation analysis. The GO classifications were performed using the WEGO online tool (Ye, 2006).

### Physical distribution of *PaMYB* genes in chromosomes

To understand the chromosomal location of *PaMYBs*, the GFF (General Feature Format) file containing the *P. aphrodite* chromosome information was downloaded from the website (http://orchidstra2.abrc.sinica.edu.tw/orchidstra2/pagenome.php) (Chao et al., 2018). MapInspect software was used to visualize physical location information. DNAMAN was used to compare the similarity of the *PaR2R3-MYB* cluster.

### Bioinformatics analysis of *R2R3-MYB* genes expression in *D. officinale*

The transcriptome of *D. officinale* were downloaded from the NCBI SRA database with the accession number PRJNA348403. Clean reads were obtained by removing the low-quality base calls (Q<20) using FASTX-toolkit (http://hannonlab.cshl.edu/fastx_toolkit), then mapped to the reference genome using TopHat2 software with the default parameters and assembled using Cufflinks software (Kim et al., 2013; Trapnell et al., 2012). The expression profiles were presented using MeV 4.9 with Pearson correlation using average linkage clustering.

To verify the transcriptome data, 20 genes were selected to perform qRT-PCR analysis. Three biological repeats were conducted. The average data was used to construct heatmap by MeV 4.9 with Pearson correlation using average linkage clustering.

A co-expression network of 20 *DoMYB* genes in response to five different treatments was constructed based on the Pearson correlation coefficients (PCCs). It was better to understand the topological relationships between stress-responsive *DoMYB* genes. All of the available gene expression data were averaged, and the PCC was calculated between any pair of 20 genes (Tang et al., 2013). All 20 genes were selected to evaluate a co-expression network based on the PCCs at a 0.05 significance level (*p*-value) using Cytoscape 3.3 (http://www.cytoscape.org).

### Expression analysis *R2R3-MYB* genes under different treatments in *D. officinale*

One-year-old plantlets of *D. officinale* were cultivated in an incubator at 25 °C with 12 h light and 12 h dark. The protocorms of *D. officinale* were cultivated at 25 °C in the dark. For the stress treatment, an equal mass protocorm of the same period was placed in MS liquid medium with an additional 300 mM NaCl, 300 mM mannitol and 100 µM abscisic acid (ABA), respectively. Then, protocorms with the same quality were collected at 0, 1, 2, 3, 4, and 7 d for further experiment. Moreover, for the treatment with hormone, the protocorms with the same size in the same period were cultivated in MS liquid medium additionally containing 100 µM methyl jasmonate (MeJA) and 100 µM salicylic acid (SA), respectively. The protocorms were collected at 0, 2, 4, 8, 24, 48 and 72 h. Protocorms, 0 h cultivated in a different medium, were selected as controls, respectively. Each experiment was repeated three times.

Total RNA was extracted by using RNAprep Pure Plant Kit (Tiangen, China). Primer Premier 5.0 was used to design the corresponding primers of *DoMYB* (Table S4). The gene *β*-actin was selected as an internal reference (GeneBank ID: JX294908). The qPCR system consisted of 12.5 µl of SYBR® Premix Ex TaqTM II, 2 µl of cDNA, 2 µl primers and 8.5 µl of ddH_2_O. The PCR reaction procedure was as follows: 95 °C for 30 s, 95 °C for 5 s, 60 °C for 30 s, 40 cycles (Jin et al., 2013). The relative expression level of genes was calculated using the 2^−ΔΔ*Ct*^ method (Livak & Schmittgen, 2001). Three biological repeats were conducted.

## Results

### Phylogenetic analysis and classification of the *R2R3-MYB* genes from *D. officinale* and *P. aphrodite*

**Figure 1:**
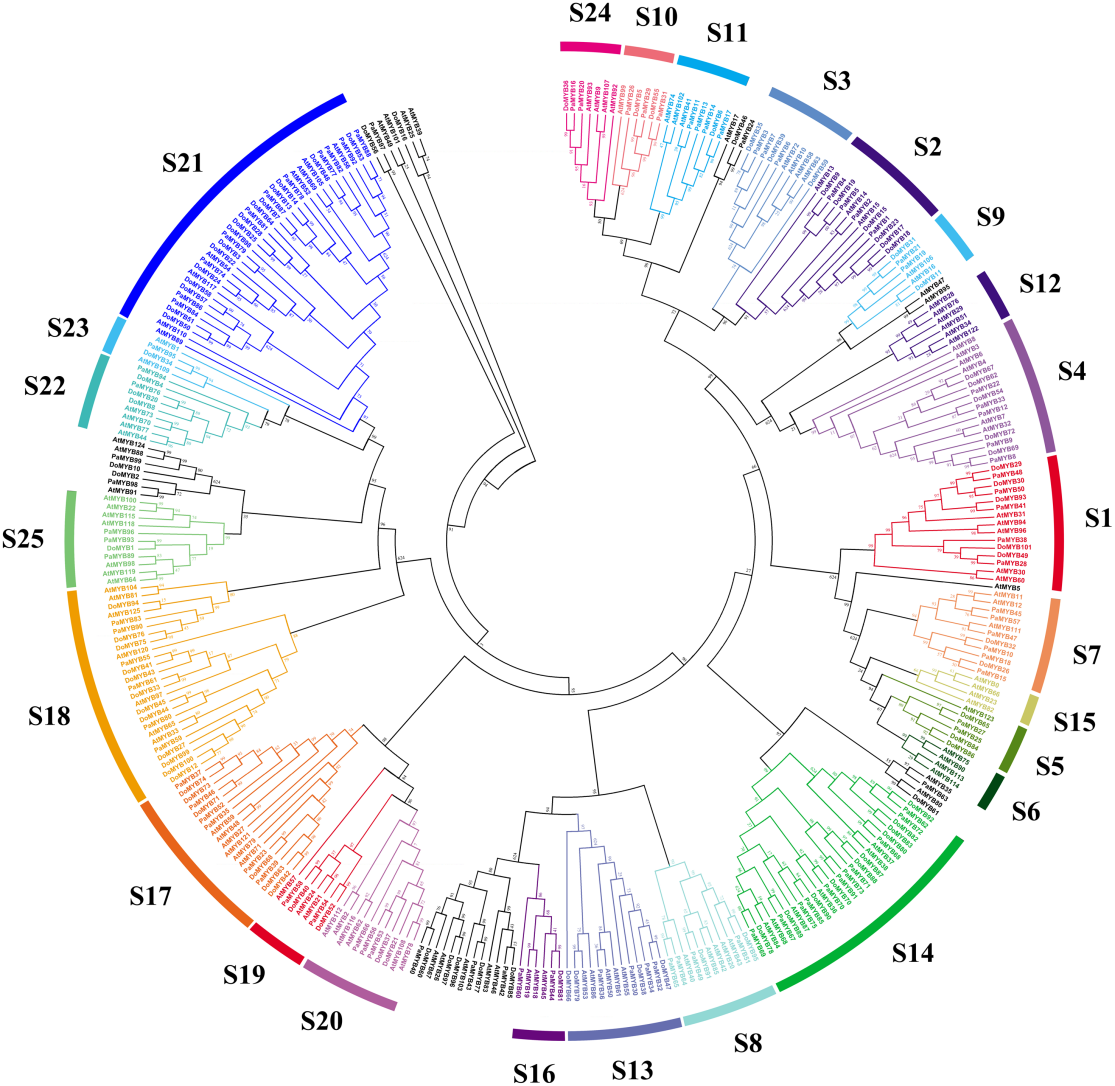
Phylogenetic analysis of R2R3-MYB proteins from *Dendrobium officinale* and *Phalaenopsis aphrodite*. In total, 101 R2R3-MYB proteins from *D. officinale*, 99 R2R3-MYB proteins from *P. aphrodite* and 126 R2R3-MYB proteins from *A. thaliana* were selected to construct the neighbor-joining tree with 1,000 bootstraps. S1-S25 indicated the divided subfamilies according to the categories of R2R3-MYB proteins in *A. thaliana*.

As was shown in Fig. 1, total of 101 *DoMYBs* and 99 *PaMYBs* were identified (Table S1). Then, 126 *R2R3-MYB* genes from *Arabidopsis* (AtMYBs) were selected as references to construct the neighbor-joining tree. The results showed that all identified *R2R3-MYB* genes could be divided into 22 subgroups with the number of members ranging from 1 to 16. Of 22 subgroups, the subfamily S21 contained the most members of *R2R3-MYB* genes with 16 *DoMYBs* and 11 *PaMYBs*, followed by S14 subfamily with 10 *DoMYBs* and 11 *PaMYBs*. The subfamily S18 contained 12 *DoMYBs* and 6 *PaMYBs*. Interestingly, nine subfamilies, including S1, S3, S4, S9, S13, S16, S17, S19, and S23, contained the same numbers of *R2R3-MYBs* from both *D. officinale* and *P. aphrodite*. For example, both six members of *DoMYBs* and *PaMYBs* were divided into S17 subfamily. S1 and S4 subfamilies included both five *R2R3-MYBs* for *D. officinale* and *P. aphrodite*. The subfamily S13 contained four *R2R3-MYBs* from both *D. officinale* and *P. aphrodite*. Otherwise, no *R2R3-MYB* genes were classified into the subfamilies S6, S12, and S15 from the two Orchidaceae species. So, the distributions of most *R2R3-MYB* genes were similar between *D. officinale* and *P. aphrodite*.

### Gene structure analysis of the *R2R3-MYB* protein from *D. officinale* and *P. aphrodite*

**Figure 2:**
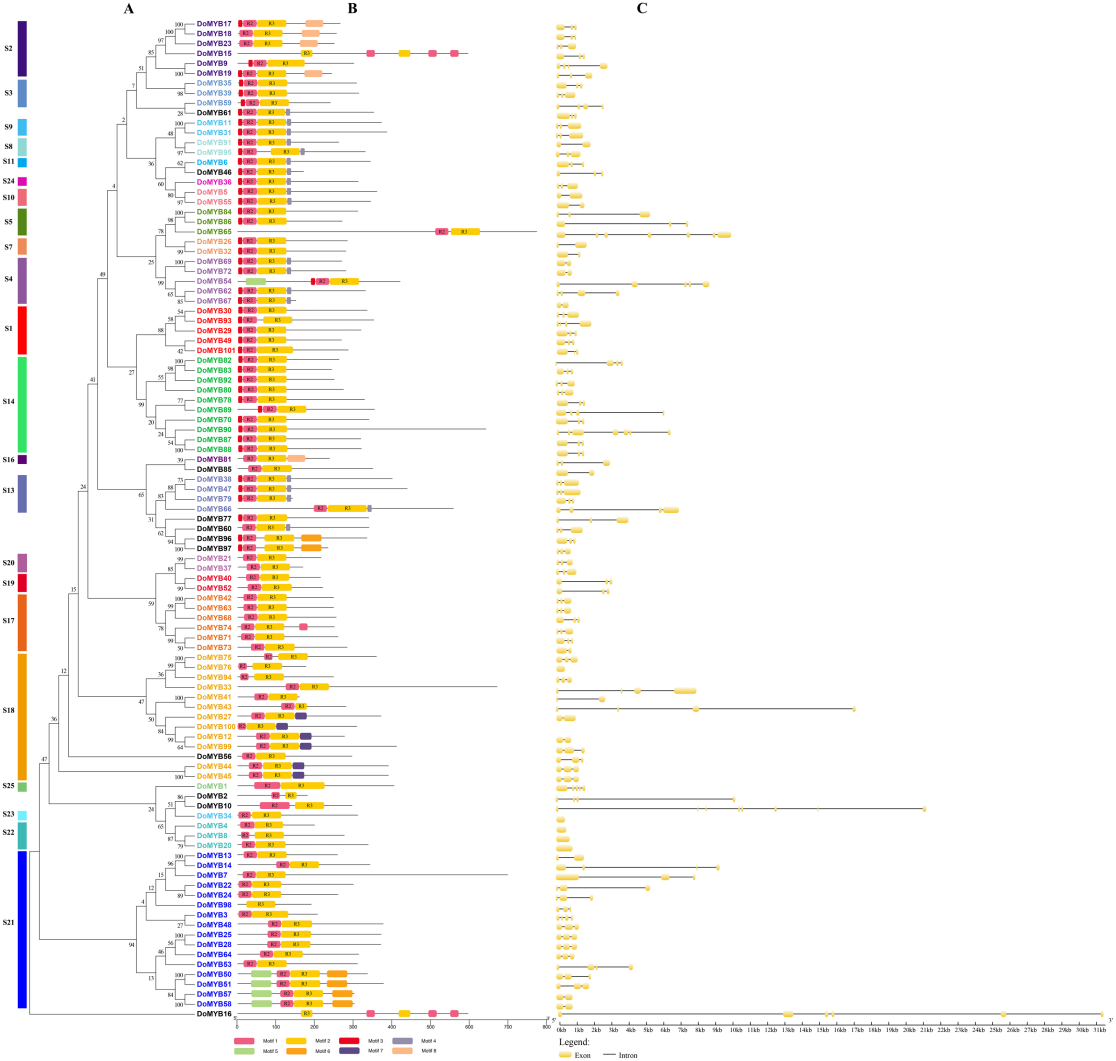
Phylogenetic relationships, intron pattern, and architecture of conserved protein motifs in R2R3-MYB proteins from *D. officinale*. (A) The phylogenetic tree constructed with 101 R2R3-MYB proteins from *D.officinale.* S1-S25 indicated the divided subfamilies. (B) Architecture of conserved protein motifs in different subfamilies. The colored boxes indicated the different motifs as listed at the bottom of the figure. (C) The predicted exon-intron structures. The yellow boxes and black lines exhibited exons and introns, respectively.

The R2R3-MYB protein sequences of *D. officinale* and *P. aphrodite* were used to create a phylogenetic tree using MEGA5.2 software (Fig. 2A and Fig. S1A). The conserved motifs were identified using the MEME tool. The results showed that most of R2R3-MYB proteins in the same subfamily contained similar motifs, which futher verifying the closeness of their evolutionary relationship with the phylogenetic tree (Fig. 2B and Fig. S1B). Nevertheless, motif 3, which didn’t belong to the conserved domain R2 or R3, was included in almost half of R2R3-MYB members in *D. officinale* and *P. aphrodite*. Otherwise, the subfamilies S4, S8, S9, S10, S11 and S13, all contained the motif 4 in *D. officinale*. Furthermore, the gene structure analysis of the R2R3-MYB genes revealed that most of these genes consisted of three exons and two introns (Fig. 2C and Fig. S1C). These results indicated that the intron patterns were highly conserved.

### Strong purifying selection analysis of the *R2R3-MYB* genes from *D. officinale* and *P. aphrodite*

To examine the selection pressure on the *R2R3-MYB* genes, 16 gene pairs between *P. aphrodite* and *D. officinale*, 19 gene pairs of *P. aphrodite* and four gene pairs of *D. officinale* with close genetic affinities were selected to calculate the Ka/Ks ratio. As a result, of 16 gene pairs between two species, 87% showed a rate less than 0.3. Of the 19 gene pairs from *D. officinale*, 79% exhibited a Ka/Ks ratio between 0 and 0.5, and only four gene pairs scored a Ka/Ks ratio greater than 0.5. Moreover, the Ka/Ks ratios of the four gene pairs from P. aphrodite were between 0 and 1 (Table S2). These results implied that most of the R2R3-MYB gene pairs from *D. officinale* and *P. aphrodite* mainly evolved under the influence of purifying selection.

### Gene ontology annotation and R domain characteristics analysis the *R2R3-MYB* genes from *D. officinale* and *P. aphrodite*

The functions of the R2R3-MYB proteins were predicted by GO annotation analysis. Based on amino acid similarity, the R2R3-MYB proteins from two Orchidaceae species were classified into three categories: biological process (BP), cellular component (CC), and molecular function (MF) (Table S5, Fig. S2). The majority of the R2R3-MYB proteins from *D. officinale* and *P. aphrodite* were enriched in two categories: biological process and molecular function. Only a small portion of the R2R3-MYB proteins was enriched in the cellular component (Fig. S2AC).

**Figure 3:**
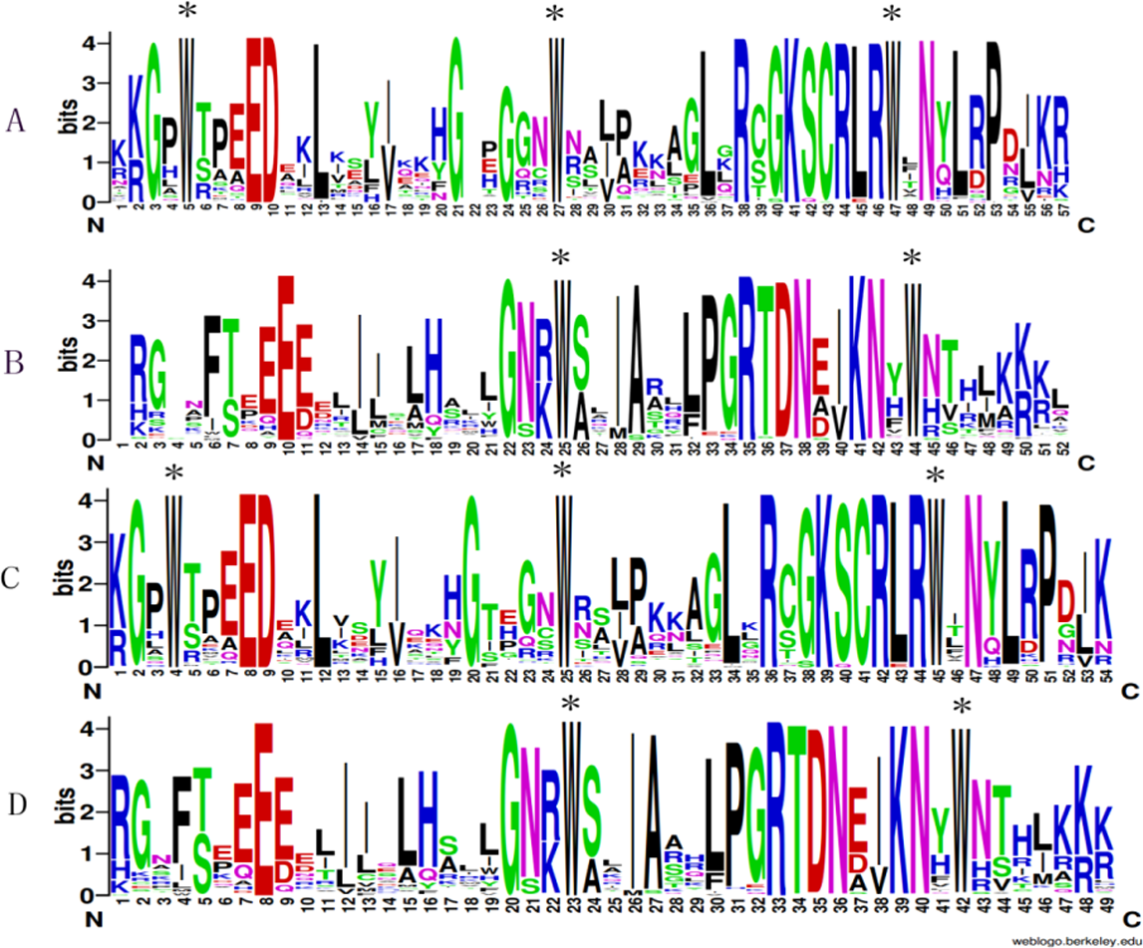
Sequence logos of the R2 (A&C) and R3 (B&D) MYB repeats. A & B respectively indicate the R2 and R3 logos based on multiple alignment analysis of 101 DoR2R3-MYB proteins. C & D indicate the R2 and R3 logos of 99 PaR2R3-MYB proteins. * indicate typical conserved Trp residues in the MYB domain.

To analyze the presence of conserved sequences at particular positions, WebLogo was used to generate sequence logos. As is shown in Fig. 3, A and B indicated the repeats of the R2 and R3 sequences of *D. officinale*, respectively. C and D showed the repeats of the R2 and R3 sequences of *P. aphrodite*, respectively. These results indicated that most amino acids are conservatively presented in the R2 and R3 repeats between two Orchidaceae species (Fig. 3). Otherwise, some amino acids are more conservative. For example, tryptophan was identified at the sites approximately per every 18 amino acids. There were three tryptophans in R2 and two in R3.

### Chromosomal location analysis of the *PaMYB* genes

To study the relationship between genetic divergence and gene duplication within the *R2R3-MYB* gene family in *P. aphrodite*, the locations and distribution of the *R2R3-MYB* genes were analyzed. As a result, of the 99 *PaMYB* genes, 85 genes were mapped and distributed in 17 chromosomes of *P. aphrodite*. The distribution of genes was uneven. Chromosome 9 contained the most *R2R3-MYB* genes (10 *PaMYB* genes), followed by chromosome 18, which contained 9 *PaMYB* genes. The other chromosomes contained *R2R3-MYB* genes, ranging from 1 to 8, respectively (Fig. S3). In addition, 33 pairs of *R2R3-MYB* proteins were isolated with similar sequences by using sequence alignment in DNAMAN (Table S6). Of these proteins, two pairs were proved to be segmental duplication for their high similarity values (PaMYB64/PaMYB65, PaMYB73/PaMYB91). PaMYB64 and PaMYB65 have an identical sequence with the same position on chromosome 9 (Table S6). PaMYB73 and PaMYB91 showed a high similarity value of 87.25% (Table S6).

### Expression analysis of *DoMYB* genes in different tissues

**Figure 4:**
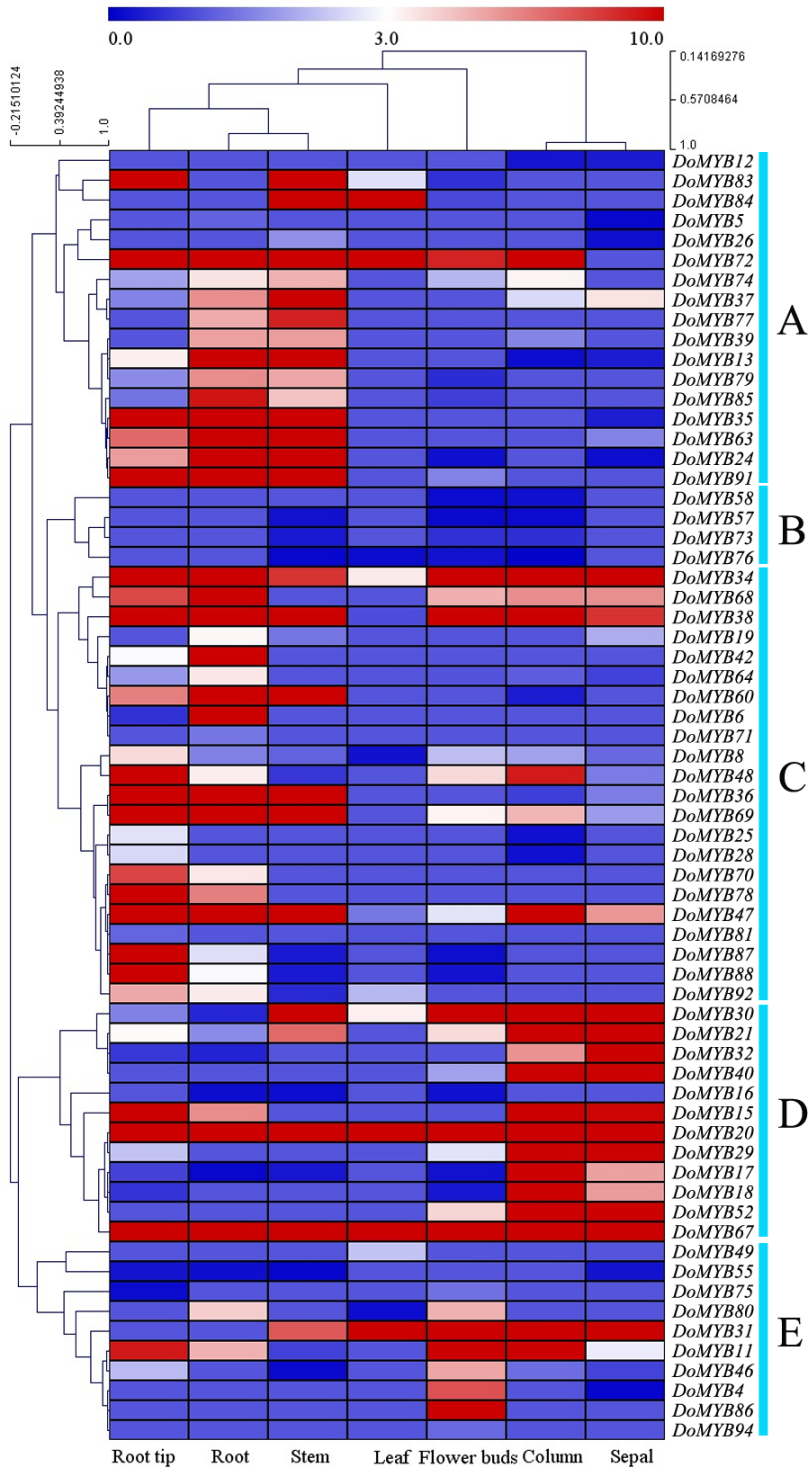
RNA-seq analysis of R2R3-MYB transcription factors in *D. officinale*. The heatmap was generated using R gplots package, and the FPKM values of *D. officinale* genes were evaluated and normalized based on RNA-seq data from NCBI SRA database. Differential expression pattern of 65 annotated R2R3-MYBs in various tissues,including root tips (RT), roots (RO), stems (ST), leaves (LE), flower buds (FB),columns (CO), and sepals (SP). The expression values are available in Table S3.

The transcriptome data of seven tissues including root tips (RT), roots (RO), stems (ST), leaves (LE), flower buds (FB), columns (CO), and sepals (SP) were downloaded from the NCBI SRA (Sequence Read Archives) database. 65 of 101 *DoMYB* genes were detected in the transcriptome analysis (Fig. 4). They were clustered into five groups (A–E). Among those groups, 17 genes were classified in group A with relatively high expression levels in roots and/or stems. Four genes in group B displayed low expression levels in various tissues. Out of 65 genes, 22 genes were clustered in group C. They had the highest transcript abundance in the roots and/or root tips. All 12 genes in group D exhibited a high expression levels in columns and/or sepals. The remaining ten genes were all in group E. They had higher expression in flower buds if compared with that in other tissues. Moreover, several genes showed tissue-specific expression patterns. For example, seven genes were only expressed in flowers (*DoMYB4*, *DoMYB12*, *DoMYB40*, *DoMYB52*, *DoMYB58*, *DoMYB86*, *DoMYB94*), three genes were only expressed in roots (*DoMYB6*, *DoMYB78*, *DoMYB81*) (Table S3).

**Figure 5:**
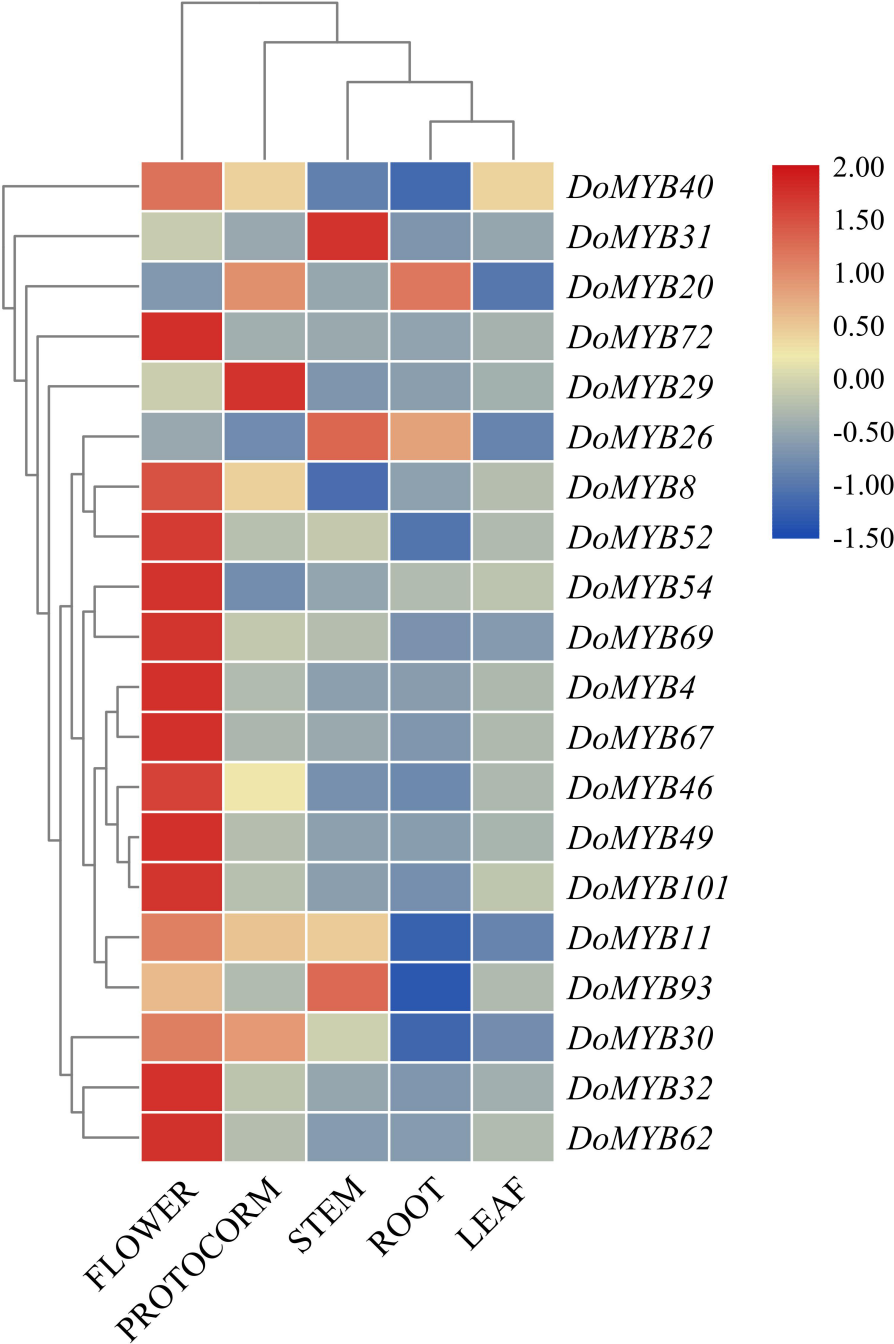
qRT-PCR validation of R2R3-MYB transcription factor genes of *D. officinale* in different tissues. Twenty genes were selected to perform qRT-PCR analysis to verify the transcriptome data. Three biological repeats were conducted. The average data were used to construct heatmap by MeV 4.9 with Pearson correlation using average linkage clustering.

**Figure 6:**
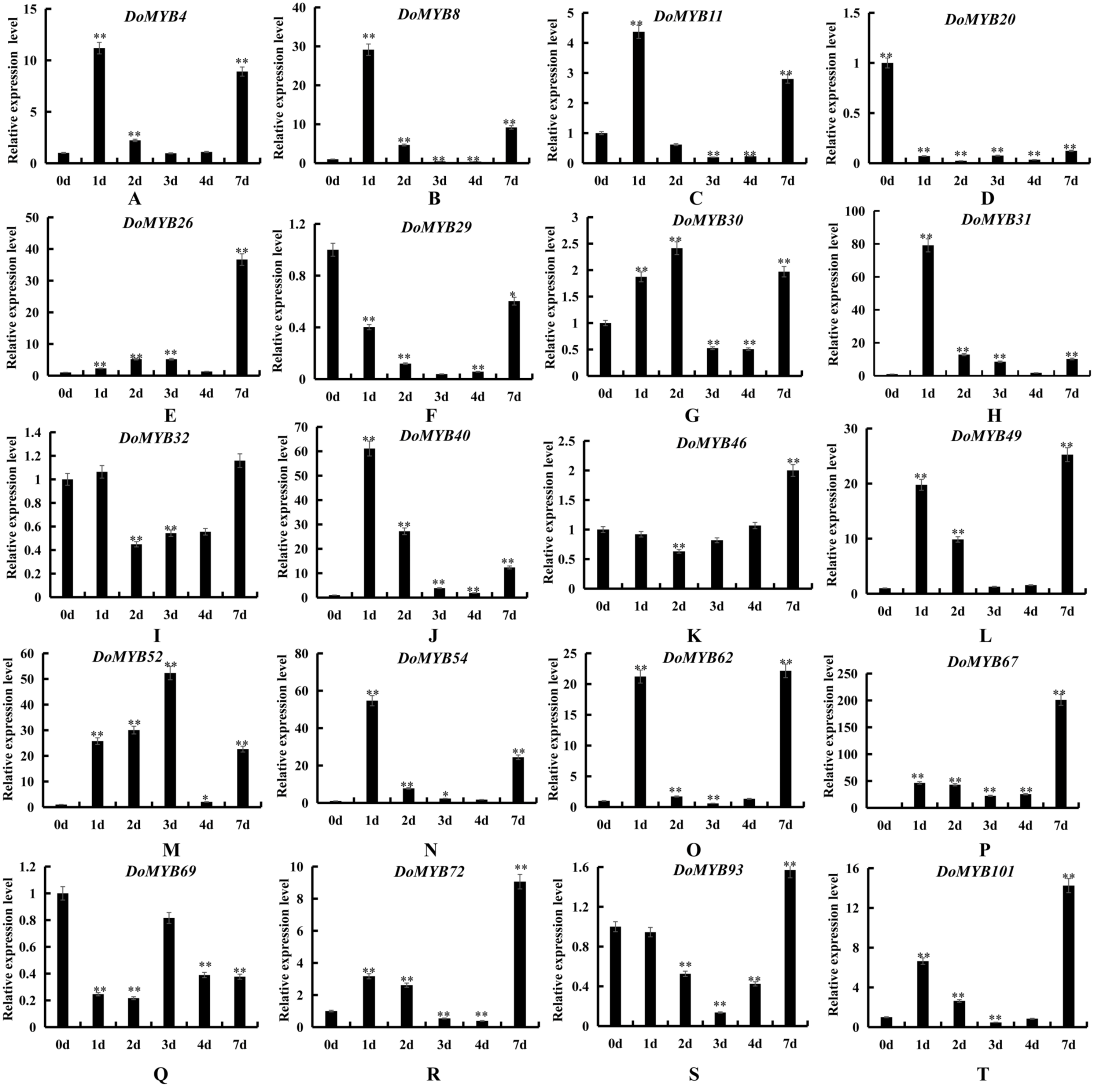
Expression patterns of the *DoMYB* genes in response to drought treatment. The relative expression levels of 20 *DoMYB* genes were examined by qRT-PCR and normalized to the reference gene *β*-actin. Every experiment had three biological repeats. The *x*-axis indicated the time course of each stress treatment, and the *y*-axis represented the relative expression level. Mean values and standard deviations (SDs) indicated by error bars. ** significant difference (*P* < 0.01), *significant difference at *P* < 0.05. (A) *DoMYB4*, (B) *DoMYB8*, (C) *DoMYB11*, (D) *DoMYB20*, (E) *DoMYB26*, (F) *DoMYB29*, (G) *DoMYB30*, (H) *DoMYB31*, (I) *DoMYB32*, (J) *DoMYB40*, (K) *DoMYB46*, (L) *DoMYB49*, (M) *DoMYB52*, (N) *DoMYB54*, (O) *DoMYB62*, (P) *DoMYB67*, (Q) *DoMYB69*, (I) *DoMYB72*, (S) *DoMYB93*, (T) *DoMYB101*.

**Figure 7:**
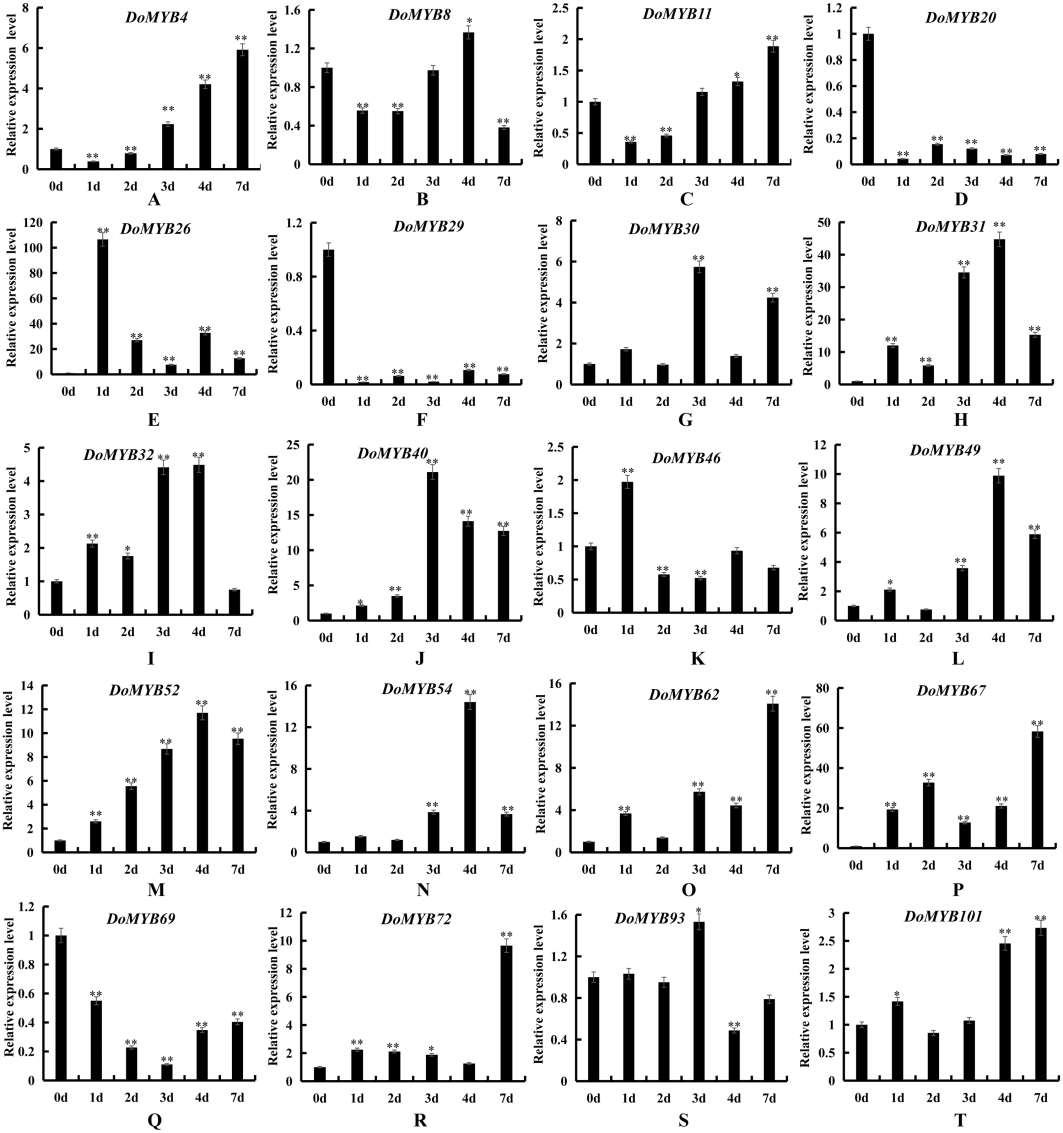
Expression patterns of the *DoMYB* genes under salt treatment. The relative expression level of 20 *DoMYB* genes was evaluated by qRT-PCR. The gene *β*-actin was selected as a reference. Every experiment had three biological repeats. The *x*-axis indicated the time course of each stress treatment, and the *y*-axis represented the relative expression level. Mean values and standard deviations (SDs) indicated by error bars. ** significant difference (*P* < 0.01), * significant difference at *P* < 0.05. (A) *DoMYB4*, (B) *DoMYB8*, (C) *DoMYB11*, (D) *DoMYB20*, (E) *DoMYB26*, (F) *DoMYB29*, (G) *DoMYB30*, (H) *DoMYB31*, (I) *DoMYB32*, (J) *DoMYB40*, (K) *DoMYB46*, (L) *DoMYB49*, (M) *DoMYB52*, (N) *DoMYB54*, (O) *DoMYB62*, (P) *DoMYB67*, (Q) *DoMYB69*, (I) *DoMYB72*, (S) *DoMYB93*, (T) *DoMYB101*.

*D. officinale* is a valuable medicinal plant for its high content of secondary metabolites, including polysaccharides, alkaloids and terpenes. Due to its strict requirements on the growth environment, it usually suffers from various biotic or abiotic stresses during its development and growth. Based on the reported functions of *R2R3-MYB* in other plants, we screened out 20 *DoMYBs* belonged to six subfamilies to study the role of *R2R3-MYB* in the regulation of secondary metabolism and the responses to abiotic stress for *D. officinale* (Stracke et al., 2007; Adato et al., 2009). Their expression patterns in different tissues (Fig. 5) and under various abiotic stresses were tested (Figs. 6 and 7). Among the five genes of the S1 subfamily, *DoMYB30, DoMYB49*, and *DoMYB101* showed higher expression levels in flowers, *DoMYB29* had higher expression levels in protocorms, and *DoMYB93* displayed higher expression levels in stems. Otherwise, five genes of the S4 subfamily showed higher expression levels in flowers. Among the S7 subfamily, DoMYB26 had the highest expression level in stems, and *DoMYB32* had the highest expression level in flowers. Among the S9 subfamily, *DoMYB11* and *DoMYB46* had higher expression levels in flowers, and *DoMYB31* had higher expression level in stems. In the S19 subfamily, *DoMYB40* and *DoMYB52* had higher expression levels in flowers. In the S22 subfamily, *DoMYB4* and *DoMYB8* had higher expression levels in flowers. Otherwise, *DoMYB20* had higher expression level in roots. These results are in accord with transcriptome data.

### Expression pattern of *DoMYB* genes in response to abiotic stress in protocorms

To explore the functions of the *R2R3-MYB* genes in *D. officinale*, the expression patterns of 20 DoMYB genes were analyzed under drought and salt stress. Multiple genes were upregulated, yet a few genes were downregulated in response to drought and salt stress. *DoMYB20, DoMYB29* and *DoMYB69* showed downregulated expression patterns under either drought or salt stress. Five *DoMYB* genes, *DoMYB31, DoMYB40, DoMYB49, DoMYB52* and *DoMYB54*, showed increased expression levels at different times under the drought treatment (Fig. 6). The expression levels of *DoMYB31, DoMYB40* and *DoMYB54*, were significantly increased after one day of treatment. The expression of *DoMYB49* increased after seven days of treatment. DoMYB52 exhibited an increased expression level after three days of treatment. Also, *DoMYB26, DoMYB31* and *DoMYB67* were identified to respond to salt stress (Fig. 7). *DoMYB26* had an increased expression level after one day of treatment. *DoMYB31* showed an increased expression level with approximately 40-fold after four days of treatment. *DoMYB67* exhibited an elevated expression level after seven days of treatment.

### Co-expression network analysis of *DoMYB* genes in different tissues and different treatments

To analyze the relationship of 20 *DoMYB* genes, a co-expression network was constructed using Cytoscape 3.3. Based on the data of the relative transcript abundance in different tissues under various kinds of treatments, a tissue co-expression network and a post-processing gene co-expression network were constructed to explore the relationship of the *MYB* genes in *D. officinale* (Fig. S4A/B). The results showed that the tissue co-expression network contained 17 nodes (*DoMYB* Genes) and 66 edges (co-expressed gene pairs), representing PCCs between tissue co-expressing events. Moreover, the processed co-expression network contained 16 nodes and 44 edges, representing the handling of PCCs between co-expression events. Each node had a different number of regulatory edges, ranging from 1 to 11.

## Discussion

MYB transcription factors (TFs) are a large gene family in plants. Previous studies have identified many MYB genes in Orchidaceae. For example, by RT-PCR, 21 *R2R3-MYB* genes have been isolated from the *Dendrobium* orchid hybrid Woo Leng. Four of these genes, *DwMYB1*, *DwMYB2*, *DwMYB8*, and *DwMYB10*, belong to typical plant *R2R3-MYB* genes (Wu, Lim & Yang, 2003). Otherwise, three *R2R3-MYB* transcription factors, including *PeMYB2*, *PeMYB11* and *PeMYB12*, have been identified and proven to be concomitant with the red color formation in flowers from *Phalaenopsis* spp (Hsu et al., 2015). Similarly, *DhMYB1* has been confirmed to be involved in the development of the conical cell shape of epidermis cells of the *Dendrobium* hybrid flower labellum (Lau et al., 2015). In this study, 101 *DoMYBs* and 99 *PaMYBs* of *R2R3-MYB* genes were identified from genomic database sequences and classified into 22 subfamilies. In contrast to the *R2R3-MYB* gene numbers found in other species, the numbers of *R2R3-MYB* genes in *D. officinale* and *P. aphrodite* were less than those in *A. thaliana* (126), *O. sativa* (109), *Z. mays* (157) and *P.trichocarpa* (192). All of the identified *R2R3-MYB* genes were divided into different subfamilies. The subfamilies S14, S18 and S21, contained the most MYBs. Nine subfamilies included the same number of *R2R3-MYB* genes from both *D. officinale* and *P. Aphrodite*. There were no *R2R3-MYB* members found in the S6, S12, and S15 *R2R3-MYB* subfamilies. These results may imply the special characteristics *R2R3-MYB* genes in two Orchidaceae species (Fig. 2).

It has been reported that several *R2R3-MYB* genes are involved in regulating responses to biotic and abiotic stresses. For example, the S1 subfamily genes *AtMYB96* and *OsMYB4* were confirmed to be involved in the drought response of plants (Seo et al., 2009; Vannini et al., 2004). *AtMYB41* and *AtMYB102* contribute to plant resistance against wounding and osmotic stress (Denekamp & Smeekens, 2003; Lippold et al., 2009). Overexpression of *AtMYB2* can be induced by dehydration and salt stress (Abe et al., 2003). *AtMYB62* participates in response to phosphate starvation (Devaiah et al., 2009). Besides, the S22 subfamily gene *AtMYB44* has been shown to be induced by drought and high salt. Overexpression of *AtMYB44* obviously enhances drought and salt tolerance in plants (Jung et al., 2007). *AmMYB1* is related to the enhancement of tolerance to NaCl stress in transgenic tobacco (Ganesan et al., 2012). In this study, 20 *R2R3-MYB* genes were tested in response to drought and salt stress treatments. Significant changes in the expression levels of all of the members of the S19 subfamily were detected (Figs. 6 and 7). For example, *DoMYB40* showed increased expression after one day of drought treatment. Then, the expression level of *DoMYB40* gradually decreased. *DoMYB52* displayed the highest expression level after three days of treatment. On the other hand, after undergoing NaCl-simulated salt stress, *DoMYB40* reached the highest expression level under treatment for three days, after which, its expression level decreased. *DoMYB52* expression increased after four days of treatment and decreased after that. These results may imply the vital role of the S19 subfamily members in response to abiotic stress.

## Conclusions

In this study, 101 and 99 *R2R3-MYB* genes were identified from the genomes of *Dendrobium officinale* and *Phalaenopsis aphrodite*. They were distributed into 22 subfamilies based on the homologous analysis. To test the function of identified *R2R3-MYB* genes, the expression pattern analysis of the *DoMYB* genes from six subfamily members in different tissues revealed that the S4 and S19 subfamily members had the highest gene expression levels in flowers. Besides, determination of expression level of *DoMYBs* verified that some of them were responsible for the stress response. These results may provide useful information for further studies of the R2R3-MYB gene family.

##  Supplemental Information

10.7717/peerj.9781/supp-1 Supplemental Information 1 Supplementary tables and figures

10.7717/peerj.9781/supp-2 Supplemental Information 2 qRT-PCR Data of DoMYB30

10.7717/peerj.9781/supp-3 Supplemental Information 3 qRT-PCR Data of DoMYB49

10.7717/peerj.9781/supp-4 Supplemental Information 4 qRT-PCR Data of DoMYB93

10.7717/peerj.9781/supp-5 Supplemental Information 5 qRT-PCR Data of DoMYB101

10.7717/peerj.9781/supp-6 Supplemental Information 6 qRT-PCR Data of DoMYB54

10.7717/peerj.9781/supp-7 Supplemental Information 7 qRT-PCR Data of DoMYB46

10.7717/peerj.9781/supp-8 Supplemental Information 8 qRT-PCR Data of DoMYB62

10.7717/peerj.9781/supp-9 Supplemental Information 9 qRT-PCR Data of DoMYB40

10.7717/peerj.9781/supp-10 Supplemental Information 10 qRT-PCR Data of DoMYB67

10.7717/peerj.9781/supp-11 Supplemental Information 11 qRT-PCR Data of DoMYB52

10.7717/peerj.9781/supp-12 Supplemental Information 12 qRT-PCR results of DoMYB69

10.7717/peerj.9781/supp-13 Supplemental Information 13 qRT-PCR Data of DoMYB29

10.7717/peerj.9781/supp-14 Supplemental Information 14 qRT-PCR Data of DoMYB4

10.7717/peerj.9781/supp-15 Supplemental Information 15 qRT-PCR Data of DoMYB72

10.7717/peerj.9781/supp-16 Supplemental Information 16 qRT-PCR Data of DoMYB8

10.7717/peerj.9781/supp-17 Supplemental Information 17 qRT-PCR Data of DoMYB26

10.7717/peerj.9781/supp-18 Supplemental Information 18 qRT-PCR Data of DoMYB20

10.7717/peerj.9781/supp-19 Supplemental Information 19 qRT-PCR Data of DoMYB32

10.7717/peerj.9781/supp-20 Supplemental Information 20 qRT-PCR Data of DoMYB11

10.7717/peerj.9781/supp-21 Supplemental Information 21 qRT-PCR Data of DoMYB31

## Additional Information and Declarations

### Data Availability

The following information was supplied regarding data availability: The raw measurements are available in the Supplemental Files.
