# Supplementary material for: Genome-wide identification and expression analyses of R2R3-MYB transcription factor genes from two Orchid species
Source: PeerJ. 2020 Sep 1;8:e9781. doi: 10.7717/peerj.9781 (PMC7473048; doi:10.7717/peerj.9781)
Supplement: Supplemental Information 1 [file peerj-08-9781-s001.docx]

**Genome-wide identification and expression analyses of R2R3-MYB transcription factor genes from two Orchid species**

Honghong Fan^†1^, Manli Cui^†1^, Ninghong Li^1^, Xujuan Li^1^, Yuxuan Liang^2^, Lin Liu^1^, Yongping Cai*^1^, Yi Lin*^1^

1. School of Life Sciences, Anhui Agricultural University, Hefei 230036, China;
2. Faculty of Forestry, University of British Columbia, Vancouver V6T1Z4, Canada;

hhfan0551@126.com (H.F.);1289121655@qq.com (M.C.); [lnh0411@163.com(N.L.)](mailto:lnh0411@163.com(N.L.))

lxj152567@163.com (X.L.); liulin2018@ahau.edu.cn (L.L.).

*Correspondence:swkx12@ahau.edu.cn (Yongping Cai); [linyi1957@126.com](mailto:linyi320722@163.com) (Yi Lin)

Tel: +86-551-65786137 (Yongping Cai); +86-551-65786340 (Yi Lin)

† These authors contributed equally to this work.

**Supplementary Table S1. The correspondence between gene serial number and nomenclature**

| ***Dendrobium officinale*** | | ***Phalaenopsis aphrodite*** | |
| --- | --- | --- | --- |
| DoMYB1 | Dendrobium_GLEAN_10034968 | PaMYB1 | PAXXG290370-mRNA1 gene=PAXXG290370 |
| DoMYB2 | Dendrobium_GLEAN_10010315 | PaMYB2 | PAXXG094110-mRNA1 gene=PAXXG094110 |
| DoMYB3 | Dendrobium_GLEAN_10045399 | PaMYB3 | PAXXG065970-mRNA1 gene=PAXXG065970 |
| DoMYB4 | Dendrobium_GLEAN_10008951 | PaMYB4 | PAXXG114410-mRNA1 gene=PAXXG114410 |
| DoMYB5 | Dendrobium_GLEAN_10095503 | PaMYB5 | PAXXG076770-mRNA1 gene=PAXXG076770 |
| DoMYB6 | Dendrobium_GLEAN_10020399 | PaMYB6 | PAXXG156600-mRNA1 gene=PAXXG156600 |
| DoMYB7 | Dendrobium_GLEAN_10019161 | PaMYB7 | PAXXG120810-mRNA1 gene=PAXXG120810 |
| DoMYB8 | Dendrobium_GLEAN_10079201 | PaMYB8 | PAXXG301890-mRNA1 gene=PAXXG301890 |
| DoMYB9 | Dendrobium_GLEAN_10111887 | PaMYB9 | PAXXG216740-mRNA1 gene=PAXXG216740 |
| DoMYB10 | Dendrobium_GLEAN_10115450 | PaMYB10 | PAXXG124400-mRNA1 gene=PAXXG124400 |
| DoMYB11 | Dendrobium_GLEAN_10080949 | PaMYB11 | PAXXG212240-mRNA1 gene=PAXXG212240 |
| DoMYB12 | Dendrobium_GLEAN_10134249 | PaMYB12 | PAXXG112540-mRNA1 gene=PAXXG112540 |
| DoMYB13 | Dendrobium_GLEAN_10054149 | PaMYB13 | PAXXG212220-mRNA1 gene=PAXXG212220 |
| DoMYB14 | Dendrobium_GLEAN_10060515 | PaMYB14 | PAXXG199350-mRNA1 gene=PAXXG199350 |
| DoMYB15 | Dendrobium_GLEAN_10085809 | PaMYB15 | PAXXG094660-mRNA1 gene=PAXXG094660 |
| DoMYB16 | PEQU_28089-D1 | PaMYB16 | PAXXG313220-mRNA1 gene=PAXXG313220 |
| DoMYB17 | Dendrobium_GLEAN_10044611 | PaMYB17 | PAXXG212230-mRNA1 gene=PAXXG212230 |
| DoMYB18 | Dendrobium_GLEAN_10044608 | PaMYB18 | PAXXG076310-mRNA1 gene=PAXXG076310 |
| DoMYB19 | Dendrobium_GLEAN_10061191 | PaMYB19 | PAXXG029600-mRNA1 gene=PAXXG029600 |
| DoMYB20 | Dendrobium_GLEAN_10088580 | PaMYB20 | PAXXG016050-mRNA1 gene=PAXXG016050 |
| DoMYB21 | Dendrobium_GLEAN_10130524 | PaMYB21 | PAXXG123420-mRNA1 gene=PAXXG123420 |
| DoMYB22 | Dendrobium_GLEAN_10025992 | PaMYB22 | PAXXG071610-mRNA1 gene=PAXXG071610 |
| DoMYB23 | Dendrobium_GLEAN_10046260 | PaMYB23 | PAXXG064080-mRNA1 gene=PAXXG064080 |
| DoMYB24 | Dendrobium_GLEAN_10126119 | PaMYB24 | PAXXG173940-mRNA1 gene=PAXXG173940 |
| DoMYB25 | Dendrobium_GLEAN_10012569 | PaMYB25 | PAXXG012280-mRNA1 gene=PAXXG012280 |
| DoMYB26 | Dendrobium_GLEAN_10037242 | PaMYB26 | PAXXG120700-mRNA1 gene=PAXXG120700 |
| DoMYB27 | Dendrobium_GLEAN_10052127 | PaMYB27 | PAXXG057720-mRNA1 gene=PAXXG057720 |
| DoMYB28 | Dendrobium_GLEAN_10058943 | PaMYB28 | PAXXG030620-mRNA1 gene=PAXXG030620 |
| DoMYB29 | Dendrobium_GLEAN_10138752 | PaMYB29 | PAXXG156540-mRNA1 gene=PAXXG156540 |
| DoMYB30 | Dendrobium_GLEAN_10013815 | PaMYB30 | PAXXG052550-mRNA1 gene=PAXXG052550 |
| DoMYB31 | Dendrobium_GLEAN_10066557 | PaMYB31 | PAXXG000780-mRNA1 gene=PAXXG000780 |
| DoMYB32 | Dendrobium_GLEAN_10087962 | PaMYB32 | PAXXG221140-mRNA1 gene=PAXXG221140 |
| DoMYB33 | Dendrobium_GLEAN_10025962 | PaMYB33 | PAXXG035800-mRNA1 gene=PAXXG035800 |
| DoMYB34 | Dendrobium_GLEAN_10065189 | PaMYB34 | PAXXG289520-mRNA1 gene=PAXXG289520 |
| DoMYB35 | Dendrobium_GLEAN_10095992 | PaMYB35 | PAXXG134010-mRNA1 gene=PAXXG134010 |
| DoMYB36 | Dendrobium_GLEAN_10025294 | PaMYB36 | PAXXG032710-mRNA1 gene=PAXXG032710 |
| DoMYB37 | Dendrobium_GLEAN_10062636 | PaMYB37 | PAXXG101860-mRNA1 gene=PAXXG101860 |
| DoMYB38 | Dendrobium_GLEAN_10046758 | PaMYB38 | PAXXG086650-mRNA1 gene=PAXXG086650 |
| DoMYB39 | Dendrobium_GLEAN_10047184 | PaMYB39 | PAXXG058610-mRNA1 gene=PAXXG058610 |
| DoMYB40 | Dendrobium_GLEAN_10117807 | PaMYB40 | PAXXG032580-mRNA1 gene=PAXXG032580 |
| DoMYB41 | Dendrobium_GLEAN_10008429 | PaMYB41 | PAXXG185380-mRNA1 gene=PAXXG185380 |
| DoMYB42 | Dendrobium_GLEAN_10068757 | PaMYB42 | PAXXG050100-mRNA1 gene=PAXXG050100 |
| DoMYB43 | Dendrobium_GLEAN_10089114 | PaMYB43 | PAXXG284530-mRNA1 gene=PAXXG284530 |
| DoMYB44 | Dendrobium_GLEAN_10099669 | PaMYB44 | PAXXG043380-mRNA1 gene=PAXXG043380 |
| DoMYB45 | Dendrobium_GLEAN_10099668 | PaMYB45 | PAXXG165560-mRNA1 gene=PAXXG165560 |
| DoMYB46 | Dendrobium_GLEAN_10144161 | PaMYB46 | PAXXG077510-mRNA1 gene=PAXXG077510 |
| DoMYB47 | Dendrobium_GLEAN_10018529 | PaMYB47 | PAXXG188610-mRNA1 gene=PAXXG188610 |
| DoMYB48 | Dendrobium_GLEAN_10082065 | PaMYB48 | PAXXG016570-mRNA1 gene=PAXXG016570 |
| DoMYB49 | Dendrobium_GLEAN_10090775 | PaMYB49 | PAXXG070520-mRNA1 gene=PAXXG070520 |
| DoMYB50 | Dendrobium_GLEAN_10009978 | PaMYB50 | PAXXG185930-mRNA1 gene=PAXXG185930 |
| DoMYB51 | Dendrobium_GLEAN_10024584 | PaMYB51 | PAXXG049360-mRNA1 gene=PAXXG049360 |
| DoMYB52 | Dendrobium_GLEAN_10031461 | PaMYB52 | PAXXG066530-mRNA1 gene=PAXXG066530 |
| DoMYB53 | Dendrobium_GLEAN_10110685 | PaMYB53 | PAXXG319690-mRNA1 gene=PAXXG319690 |
| DoMYB54 | Dendrobium_GLEAN_10121338 | PaMYB54 | PAXXG043940-mRNA1 gene=PAXXG043940 |
| DoMYB55 | Dendrobium_GLEAN_10010002 | PaMYB55 | PAXXG020210-mRNA1 gene=PAXXG020210 |
| DoMYB56 | Dendrobium_GLEAN_10021819 | PaMYB56 | PAXXG304660-mRNA1 gene=PAXXG304660 |
| DoMYB57 | Dendrobium_GLEAN_10035074 | PaMYB57 | PAXXG221190-mRNA1 gene=PAXXG221190 |
| DoMYB58 | Dendrobium_GLEAN_10049521 | PaMYB58 | PAXXG122360-mRNA1 gene=PAXXG122360 |
| DoMYB59 | Dendrobium_GLEAN_10073403 | PaMYB59 | PAXXG243400-mRNA1 gene=PAXXG243400 |
| DoMYB60 | Dendrobium_GLEAN_10110987 | PaMYB60 | PAXXG065450-mRNA1 gene=PAXXG065450 |
| DoMYB61 | Dendrobium_GLEAN_10115025 | PaMYB61 | PAXXG034640-mRNA1 gene=PAXXG034640 |
| DoMYB62 | Dendrobium_GLEAN_10137837 | PaMYB62 | PAXXG242360-mRNA1 gene=PAXXG242360 |
| DoMYB63 | Dendrobium_GLEAN_10016735 | PaMYB63 | PAXXG037080-mRNA1 gene=PAXXG037080 |
| DoMYB64 | Dendrobium_GLEAN_10031152 | PaMYB64 | PAXXG039810-mRNA1 gene=PAXXG039810 |
| DoMYB65 | Dendrobium_GLEAN_10041932 | PaMYB65 | PAXXG039790-mRNA1 gene=PAXXG039790 |
| DoMYB66 | Dendrobium_GLEAN_10047321 | PaMYB66 | PAXXG099050-mRNA1 gene=PAXXG099050 |
| DoMYB67 | PEQU_10866-D1 | PaMYB67 | PAXXG313160-mRNA1 gene=PAXXG313160 |
| DoMYB68 | Dendrobium_GLEAN_10085657 | PaMYB68 | PAXXG344790-mRNA1 gene=PAXXG344790 |
| DoMYB69 | Dendrobium_GLEAN_10111590 | PaMYB69 | PAXXG119190-mRNA1 gene=PAXXG119190 |
| DoMYB70 | Dendrobium_GLEAN_10078425 | PaMYB70 | PAXXG051890-mRNA1 gene=PAXXG051890 |
| DoMYB71 | Dendrobium_GLEAN_10036898 | PaMYB71 | PAXXG337540-mRNA1 gene=PAXXG337540 |
| DoMYB72 | Dendrobium_GLEAN_10024521 | PaMYB72 | PAXXG006110-mRNA1 gene=PAXXG006110 |
| DoMYB73 | Dendrobium_GLEAN_10024637 | PaMYB73 | PAXXG100080-mRNA1 gene=PAXXG100080 |
| DoMYB74 | Dendrobium_GLEAN_10121098 | PaMYB74 | PAXXG162210-mRNA1 gene=PAXXG162210 |
| DoMYB75 | Dendrobium_GLEAN_10035744 | PaMYB75 | PAXXG018180-mRNA1 gene=PAXXG018180 |
| DoMYB76 | Dendrobium_GLEAN_10045773 | PaMYB76 | PAXXG145640-mRNA1 gene=PAXXG145640 |
| DoMYB77 | Dendrobium_GLEAN_10142210 | PaMYB77 | PAXXG086530-mRNA1 gene=PAXXG086530 |
| DoMYB78 | Dendrobium_GLEAN_10012668 | PaMYB78 | PAXXG039520-mRNA1 gene=PAXXG039520 |
| DoMYB79 | Dendrobium_GLEAN_10033009 | PaMYB79 | PAXXG012650-mRNA1 gene=PAXXG012650 |
| DoMYB80 | Dendrobium_GLEAN_10095264 | PaMYB80 | PAXXG240250-mRNA1 gene=PAXXG240250 |
| DoMYB81 | Dendrobium_GLEAN_10086849 | PaMYB81 | PAXXG104200-mRNA1 gene=PAXXG104200 |
| DoMYB82 | Dendrobium_GLEAN_10012300 | PaMYB82 | PAXXG102390-mRNA1 gene=PAXXG102390 |
| DoMYB83 | Dendrobium_GLEAN_10012766 | PaMYB83 | PAXXG019490-mRNA1 gene=PAXXG019490 |
| DoMYB84 | Dendrobium_GLEAN_10078959 | PaMYB84 | PAXXG123320-mRNA1 gene=PAXXG123320 |
| DoMYB85 | Dendrobium_GLEAN_10045103 | PaMYB85 | PAXXG223570-mRNA1 gene=PAXXG223570 |
| DoMYB86 | Dendrobium_GLEAN_10075562 | PaMYB86 | PAXXG029670-mRNA1 gene=PAXXG029670 |
| DoMYB87 | Dendrobium_GLEAN_10052884 | PaMYB87 | PAXXG344110-mRNA1 gene=PAXXG344110 |
| DoMYB88 | Dendrobium_GLEAN_10052883 | PaMYB88 | PAXXG139190-mRNA1 gene=PAXXG139190 |
| DoMYB89 | Dendrobium_GLEAN_10080495 | PaMYB89 | PAXXG035040-mRNA1 gene=PAXXG035040 |
| DoMYB90 | Dendrobium_GLEAN_10057010 | PaMYB90 | PAXXG114460-mRNA1 gene=PAXXG114460 |
| DoMYB91 | Dendrobium_GLEAN_10066788 | PaMYB91 | PAXXG100085-mRNA1 gene=PAXXG100085 |
| DoMYB92 | Dendrobium_GLEAN_10027065 | PaMYB92 | PAXXG030750-mRNA1 gene=PAXXG030750 |
| DoMYB93 | Dendrobium_GLEAN_10086804 | PaMYB93 | PAXXG262150-mRNA1 gene=PAXXG262150 |
| DoMYB94 | Dendrobium_GLEAN_10111901 | PaMYB94 | PAXXG259710-mRNA1 gene=PAXXG259710 |
| DoMYB95 | Dendrobium_GLEAN_10051842 | PaMYB95 | PAXXG140220-mRNA1 gene=PAXXG140220 |
| DoMYB96 | Dendrobium_GLEAN_10056850 | PaMYB96 | PAXXG060180-mRNA1 gene=PAXXG060180 |
| DoMYB97 | Dendrobium_GLEAN_10113292 | PaMYB97 | PAXXG086900-mRNA1 gene=PAXXG086900 |
| DoMYB98 | Dendrobium_GLEAN_10007473 | PaMYB98 | PAXXG329710-mRNA1 gene=PAXXG329710 |
| DoMYB99 | Dendrobium_GLEAN_10134239 | PaMYB99 | PAXXG016480-mRNA1 gene=PAXXG016480 |
| DoMYB100 | Dendrobium_GLEAN_10134233 |  |  |
| DoMYB101 | Dendrobium_GLEAN_10046976 |  |  |

**Supplementary Table S2. Ka/Ks analysis of D. officinale and P. aphrodite *R2R3-MYB* Genes**

| **Seq 1** | **Seq 2** | **Ka** | **Ks** | **Ka/Ks** |
| --- | --- | --- | --- | --- |
| **Ka/Ks analysis between two species** | | | | |
| DoMYB9 | PaMYB4 | 0.1147 | 0.5096 | 0.246507629 |
| DoMYB19 | PaMYB5 | 0.1111 | 0.4653 | 0.238770686 |
| DoMYB26 | PaMYB15 | 0.1172 | 0.3973 | 0.294991191 |
| DoMYB32 | PaMYB10 | 0.0828 | 0.6514 | 0.127110838 |
| DoMYB33 | PaMYB61 | 0.2779 | 0.3118 | 0.891276459 |
| DoMYB39 | PaMYB3 | 0.1418 | 0.5793 | 0.244778181 |
| DoMYB47 | PaMYB32 | 0.0768 | 0.5238 | 0.146620848 |
| DoMYB49 | PaMYB28 | 0.042 | 0.3759 | 0.111731844 |
| DoMYB52 | PaMYB54 | 0.0872 | 0.4528 | 0.192579505 |
| DoMYB55 | PaMYB31 | 2.5103 | 2.11 | 1.18971564 |
| DoMYB60 | PaMYB40 | 0.0441 | 0.4924 | 0.089561332 |
| DoMYB70 | PaMYB70 | 0.0943 | 0.4172 | 0.226030681 |
| DoMYB71 | PaMYB46 | 0.1045 | 0.3924 | 0.266309888 |
| DoMYB74 | PaMYB37 | 0.0649 | 0.5351 | 0.121285741 |
| DoMYB77 | PaMYB43 | 0.1003 | 0.4603 | 0.217901369 |
| DoMYB95 | PaMYB51 | 0.0814 | 0.3367 | 0.241758242 |
| **Ka/Ks analysis of *D. officinale*** | | | | |
| DoMYB3 | DoMYB98 | 0.1683 | 1.0145 | 0.165894529 |
| DoMYB5 | DoMYB55 | 0.2249 | 0.8099 | 0.277688604 |
| DoMYB11 | DoMYB31 | 0.1229 | 0.6804 | 0.180629042 |
| DoMYB13 | DoMYB14 | 0 | 0 | 0 |
| DoMYB17 | DoMYB18 | 0.0035 | 0.0277 | 0.126353791 |
| DoMYB22 | DoMYB24 | 0.0759 | 0.0893 | 0.849944009 |
| DoMYB25 | DoMYB28 | 0 | 0 | 0 |
| DoMYB26 | DoMYB32 | 0.2766 | 1.0196 | 0.271282856 |
| DoMYB29 | DoMYB30 | 0.1152 | 0.7619 | 0.151200945 |
| DoMYB40 | DoMYB52 | 0.2743 | 0.2161 | 1.269319759 |
| DoMYB42 | DoMYB63 | 0.0072 | 0.0217 | 0.331797235 |
| DoMYB44 | DoMYB45 | 0.0012 | 0 | 0 |
| DoMYB50 | DoMYB51 | 0.0642 | 0.0505 | 1.271287129 |
| DoMYB57 | DoMYB58 | 0.0015 | 0 | 0 |
| DoMYB69 | DoMYB72 | 0.1358 | 1.3305 | 0.102066892 |
| DoMYB82 | DoMYB83 | 0.0114 | 0.0065 | 1.753846154 |
| DoMYB84 | DoMYB86 | 0.0568 | 0.1145 | 0.496069869 |
| DoMYB87 | DoMYB88 | 0 | 0 | 0 |
| DoMYB96 | DoMYB97 | 0.0158 | 0.0352 | 0.448863636 |
| **Ka/Ks analysis of *P. aphrodite*** | | | | |
| PaMYB4 | PaMYB5 | 0.2036 | 0.7306 | 0.278675062 |
| PaMYB10 | PaMYB18 | 0.2373 | 1.1108 | 0.213629816 |
| PaMYB11 | PaMYB13 | 0.2037 | 0.2212 | 0.920886076 |
| PaMYB64 | PaMYB65 | 0 | 0 | 0 |

**Supplementary Table S3. The FPKM values of *R2R3-MYB* Genes in *D. officinale* different tissues**

| **Gene** | **root tip** | **root** | **stem** | **leaf** | **flower buds** | **column** | **sepal** |
| --- | --- | --- | --- | --- | --- | --- | --- |
| DoMYB4 | 0 | 0 | 0 | 0 | 7.791064 | 0 | 0.07264 |
| DoMYB5 | 1.163071 | 1.019488 | 0 | 0 | 0 | 0 | 0.104882 |
| DoMYB6 | 42.227398 | 0.581679 | 0 | 0 | 0 | 0 | 0 |
| DoMYB8 | 1.517585 | 4.02296 | 1.171768 | 0.200046 | 2.230415 | 1.90697 | 1.227892 |
| DoMYB11 | 5.15396 | 9.330065 | 0.780061 | 0 | 24.575626 | 10.367327 | 2.782314 |
| DoMYB12 | 0 | 0 | 0 | 0 | 1.020884 | 0.255243 | 0.322008 |
| DoMYB13 | 19.982557 | 3.455765 | 18.687738 | 0 | 0 | 0.163337 | 0.331953 |
| DoMYB15 | 6.167905 | 16.66584 | 0 | 0 | 0 | 20.505466 | 9.861197 |
| DoMYB16 | 0.154621 | 0 | 0.163683 | 0 | 0.188973 | 0 | 0 |
| DoMYB17 | 0.092493 | 0.788293 | 0.275013 | 0 | 0.211013 | 27.853569 | 5.631164 |
| DoMYB18 | 0 | 0.603958 | 0 | 0 | 0.277992 | 23.415892 | 5.759681 |
| DoMYB19 | 3.208767 | 0 | 1.377532 | 0 | 0 | 0 | 2.026533 |
| DoMYB20 | 62.575939 | 90.855927 | 42.775696 | 21.672766 | 67.592857 | 409.996368 | 230.484146 |
| DoMYB21 | 1.6238 | 3.151571 | 7.141358 | 0 | 4.038577 | 17.528202 | 34.806602 |
| DoMYB24 | 19.01816 | 5.709451 | 12.566053 | 0 | 0.185891 | 0.993376 | 0.163961 |
| DoMYB25 | 0 | 2.654881 | 0 | 0 | 0 | 0.191889 | 0 |
| DoMYB26 | 0 | 0 | 1.694356 | 0 | 0 | 0 | 0.186112 |
| DoMYB28 | 0 | 2.530605 | 0 | 0 | 0 | 0.191889 | 0 |
| DoMYB29 | 0 | 2.301601 | 0 | 0 | 2.660169 | 83.035156 | 43.324272 |
| DoMYB30 | 0.456576 | 1.506463 | 38.205925 | 3.388831 | 12.300641 | 30.555271 | 57.603455 |
| DoMYB31 | 0 | 0 | 7.561275 | 10.478486 | 100.131409 | 31.198084 | 57.71059 |
| DoMYB32 | 0.423169 | 0.6594 | 0 | 0 | 0 | 6.019533 | 11.159463 |
| DoMYB34 | 15.623549 | 15.701364 | 8.621026 | 3.585867 | 11.677266 | 16.526257 | 10.842872 |
| DoMYB35 | 38.920853 | 16.249727 | 14.848194 | 0 | 0 | 0 | 0.341857 |
| DoMYB36 | 13.316644 | 32.052788 | 13.332479 | 0 | 0 | 0.739228 | 1.481223 |
| DoMYB37 | 6.098392 | 1.54512 | 13.377605 | 0 | 0 | 2.564878 | 3.747879 |
| DoMYB38 | 58.835899 | 41.687008 | 23.691772 | 0.9149 | 15.720546 | 12.293862 | 8.608888 |
| DoMYB39 | 5.590816 | 0 | 5.700919 | 0 | 0 | 1.560722 | 0 |
| DoMYB40 | 0 | 0 | 0 | 0 | 1.901877 | 782.346741 | 1174.143433 |
| DoMYB42 | 12.627621 | 2.962529 | 0 | 0 | 0 | 0 | 0 |
| DoMYB46 | 0 | 2.19833 | 0.118739 | 0 | 5.47461 | 1.283595 | 0.791961 |
| DoMYB47 | 15.167316 | 92.575523 | 10.59808 | 1.413621 | 2.690087 | 18.834719 | 5.856931 |
| DoMYB48 | 3.517563 | 14.812691 | 0.654229 | 0 | 4.057217 | 9.304658 | 1.455617 |
| DoMYB49 | 0 | 0 | 0 | 2.308003 | 0 | 0 | 0 |
| DoMYB52 | 0 | 0 | 0 | 0 | 4.156696 | 199.335648 | 31.243479 |
| DoMYB55 | 0.165557 | 0.242635 | 0.071034 | 0 | 0 | 0 | 0.22574 |
| DoMYB57 | 0 | 0 | 0.202339 | 0 | 0.127756 | 0.133497 | 0 |
| DoMYB58 | 0 | 0 | 0 | 0 | 0.13595 | 0.191154 | 0 |
| DoMYB60 | 64.785217 | 6.510294 | 12.617698 | 0 | 0 | 0.325527 | 0 |
| DoMYB63 | 25.528671 | 7.124429 | 11.712935 | 0 | 0 | 0 | 1.544544 |
| DoMYB64 | 3.660298 | 1.793412 | 0 | 0 | 0.985408 | 1.099025 | 0.783881 |
| DoMYB67 | 20.433294 | 32.511066 | 36.613266 | 16.603666 | 35.203663 | 75.015862 | 64.72934 |
| DoMYB68 | 13.141126 | 8.050599 | 0 | 0 | 5.195398 | 6.11262 | 6.063405 |
| DoMYB69 | 32.592529 | 53.526489 | 33.073257 | 0 | 3.351827 | 5.052813 | 1.831086 |
| DoMYB70 | 3.671577 | 8.146753 | 0 | 0 | 0 | 0 | 0 |
| DoMYB71 | 1.380353 | 0 | 0 | 0 | 0 | 0 | 0 |
| DoMYB72 | 76.654472 | 54.76194 | 54.318623 | 55.228989 | 9.091622 | 83.859894 | 0 |
| DoMYB73 | 0 | 0 | 0.297191 | 0 | 0.570398 | 0.564286 | 0 |
| DoMYB74 | 3.790415 | 1.896996 | 5.152054 | 0 | 2.144379 | 3.323601 | 0 |
| DoMYB75 | 0 | 0.131921 | 0 | 0 | 1.325071 | 0 | 0 |
| DoMYB76 | 0 | 0 | 0.083386 | 0.141838 | 0.212406 | 0.055464 | 0 |
| DoMYB77 | 5.321559 | 0 | 9.100634 | 0 | 0 | 0 | 0 |
| DoMYB78 | 6.528449 | 15.342223 | 0 | 0 | 0 | 0 | 0 |
| DoMYB79 | 6.151891 | 1.656904 | 5.436463 | 0 | 0.526 | 0 | 0 |
| DoMYB80 | 4.387292 | 0 | 0 | 0.163609 | 5.154045 | 0 | 0 |
| DoMYB81 | 0 | 1.180339 | 0 | 0 | 0 | 0 | 0 |
| DoMYB83 | 0 | 11.594481 | 25.835283 | 2.626018 | 0.565185 | 0 | 0 |
| DoMYB84 | 0 | 0 | 15.649305 | 12.342829 | 0.848185 | 0 | 0 |
| DoMYB85 | 9.45335 | 1.354379 | 4.662039 | 0 | 0.741842 | 0 | 0 |
| DoMYB86 | 0 | 0 | 0 | 0 | 18.897474 | 0 | 0 |
| DoMYB87 | 2.622146 | 15.977431 | 0.305709 | 0 | 0.171677 | 0 | 0 |
| DoMYB88 | 2.931346 | 12.157212 | 0.296686 | 0 | 0.217977 | 0 | 0 |
| DoMYB91 | 154.849472 | 61.685177 | 113.982475 | 0 | 1.541404 | 0 | 0 |
| DoMYB92 | 3.525032 | 5.373814 | 0.487152 | 2.17561 | 0 | 0 | 0 |
| DoMYB94 | 0 | 0 | 0 | 0 | 1.246839 | 0 | 0 |

**Supplementary Table S4. Primer sequences used in qPCR**

| **Subfamily classification** | **Primer name** | **Sequence (5'-3')** |
| --- | --- | --- |
| Subfamily 1 | DoMYB29-S | TGAGAATTATGGGGGAGCAAATACT |
|  | DoMYB29-AS | GAGAAGCATCATCAAGAGACAAATC |
|  | DoMYB30-S | ACCTAAGGCCAGGAATCAAGC |
|  | DoMYB30-AS | AGAAGCTATGGCTGCCCATC |
|  | DoMYB49-S | GCCATCGCCTCTTATCTTC |
|  | DoMYB49-AS | CGGCGAACTCTGGGTTTA |
|  | DoMYB93-S | AAGCTCTCTCCGAGGCCTTA |
|  | DoMYB93-AS | TTTCGGGGTGCTCTTCATCC |
|  | DoMYB101-S | TGGAACACCCACCTCAAGAA |
|  | DoMYB101-AS | GATGGGGTAGCCTCCATGAAC |
| Subfamily 4 | DoMYB54-S | TCTTGACCTCACCATAAGCCTACCT |
|  | DoMYB54-AS | CAGCTCTGCAACATATTACAGCTGC |
|  | DoMYB62-S | GGCATCGATCCCCAAACTCA |
|  | DoMYB62-AS | TCGGACGTATCGACGGTTTC |
|  | DoMYB67-S | TCACACCAACAAAGGGGCAT |
|  | DoMYB67-AS | GGCAACTCTTCCCACAACGA |
|  | DoMYB69-S | CCGGGAAGGACTGACAATGA |
|  | DoMYB69-AS | CTTGTTGCTCGTGGAGTGGA |
|  | DoMYB72-S | GGCGCTTGGACTAAGGAAGA |
|  | DoMYB72-AS | TGAGATCAGGGCGGAGGTAA |
| Subfamily 7 | DoMYB26-S | TCCGCCTCCATTCTTTGT |
|  | DoMYB26-AS | GCATTTCGCTTCATTGTTAT |
|  | DoMYB32-S | CCCGGTCGAACGGATAATGA |
|  | DoMYB32-AS | TTGGCCTTGGGGTTGCTTC |
| Subfamily 9 | DoMYB11-S | CAGCAGCACCGTTTGTAAGC |
|  | DoMYB11-AS | GCTTCATCCGACAGCATTCC |
|  | DoMYB31-S | ACGCCAGAGGAGGACCAGAA |
|  | DoMYB31-AS | CCTTGGATTCTTGGTGGC |
|  | DoMYB46-S | CAGGCAGGACTGATAACGA |
|  | DoMYB46-AS | GGGTGCGTATGTATCAGGATT |
| Subfamily 19 | DoMYB40-S | GCCTCCTGGTTTCCGTTT |
|  | DoMYB40-AS | AAACGGAAACCAGGAGGC |
|  | DoMYB52-S | ATGGTCCACGGTCCCTTCC |
|  | DoMYB52-AS | TGCCTACAGTGGAAGTATCAA |
| Subfamily 22 | DoMYB4-S | GGTTGCGTTGGTGCAATCAG |
|  | DoMYB4-AS | TACGACCAGGTAGAAGGCGA |
|  | DoMYB8-S | CGGCATCGCAGGAAAGAATG |
|  | DoMYB8-AS | GGCGATTCGGACTCCTAGTG |
|  | DoMYB20-S | CGGTGGTGCAACCAACTTTC |
|  | DoMYB20-AS | ATGGCGTTATCCGTACGACC |
| Actin | β-actin-AS | GGCCACTAGCATATAGGGAAAG |
|  | β-actin-S | TCCCAAGGCAAACAGAGAAA |

**Supplementary Table S5. Blast2Go annotation details of MYB protein sequences**


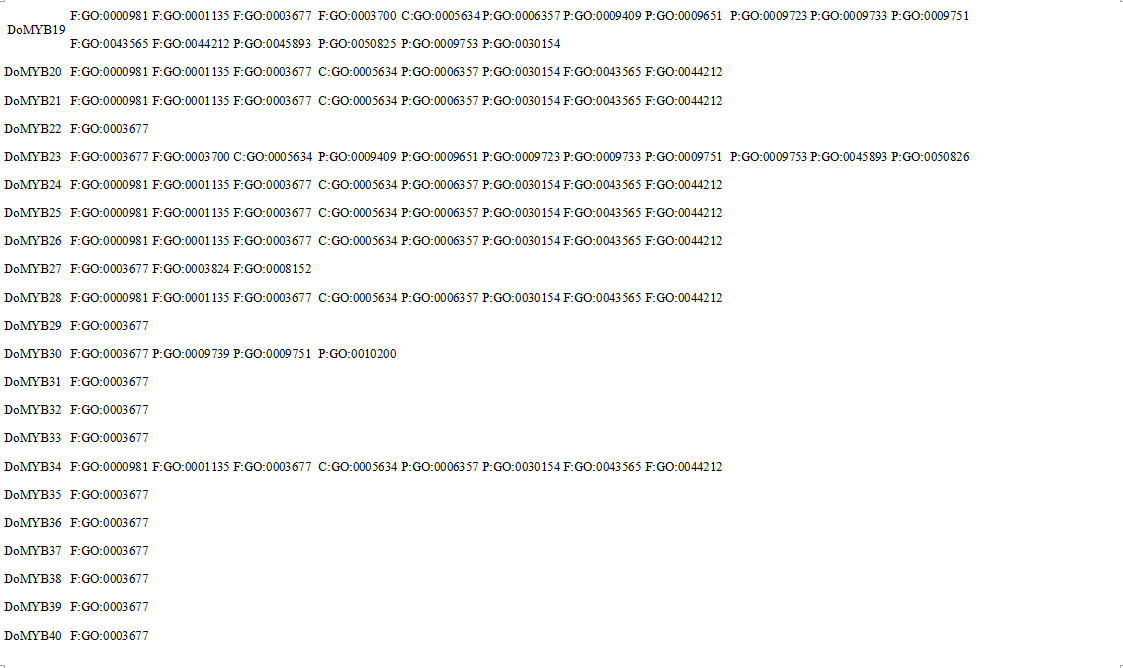

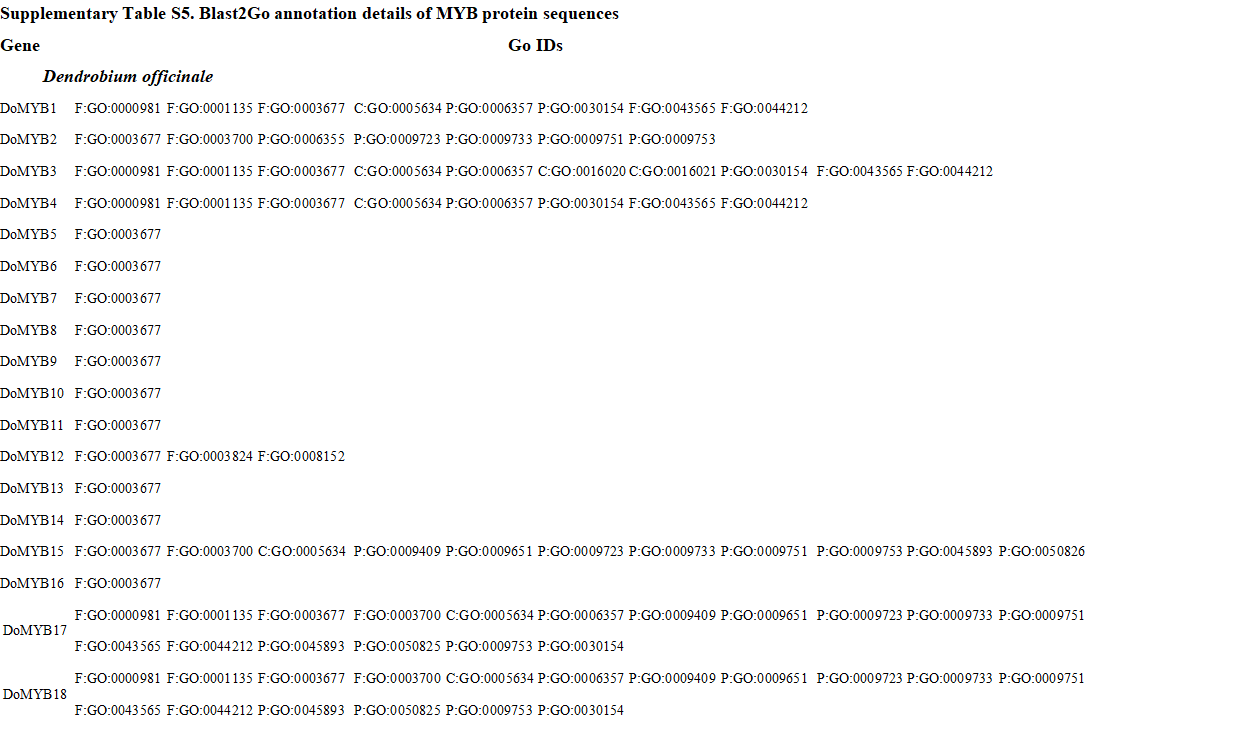


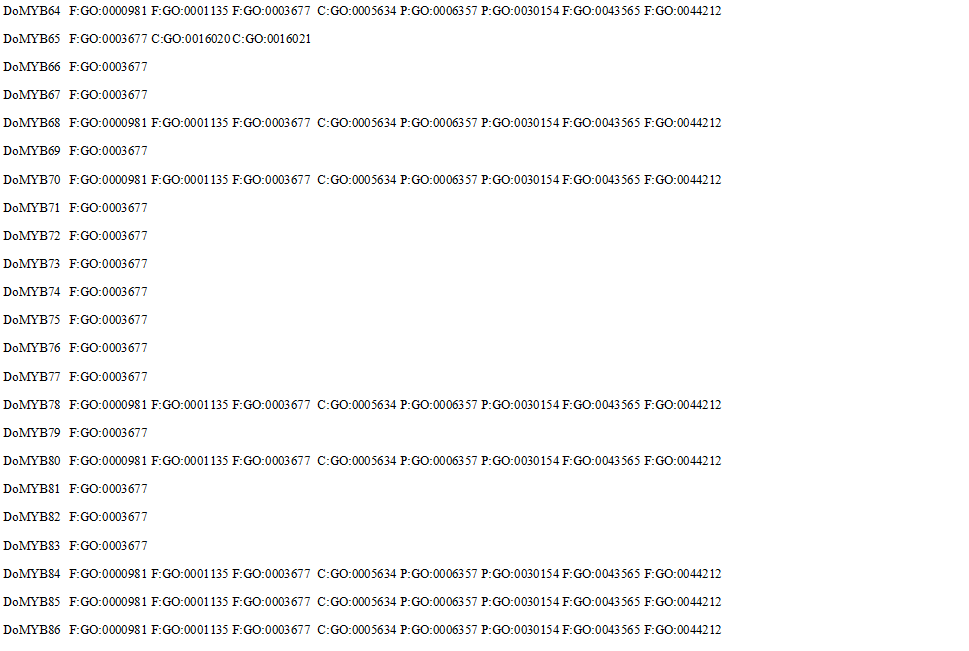


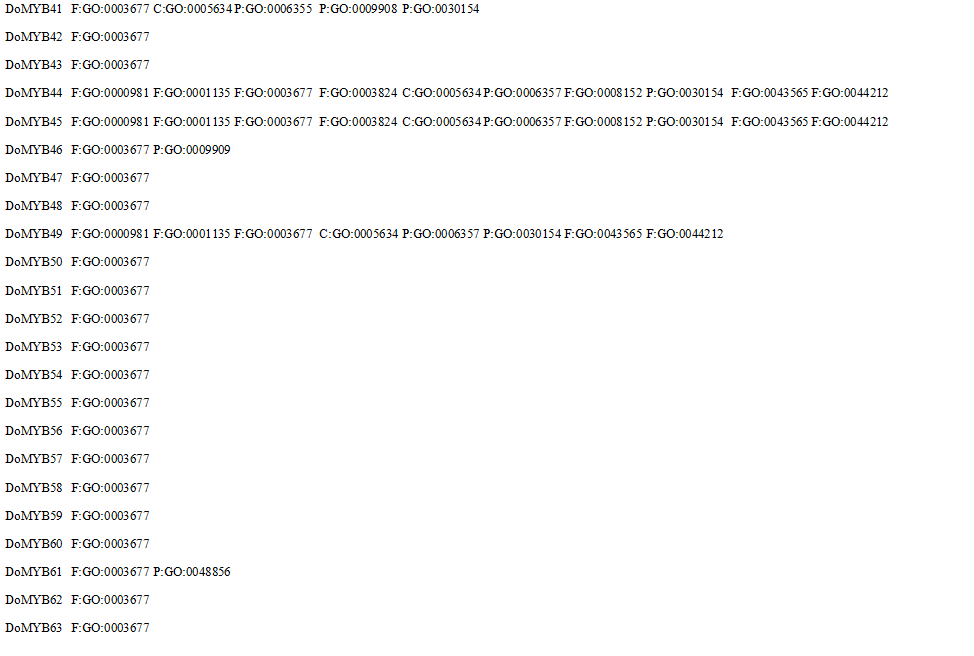


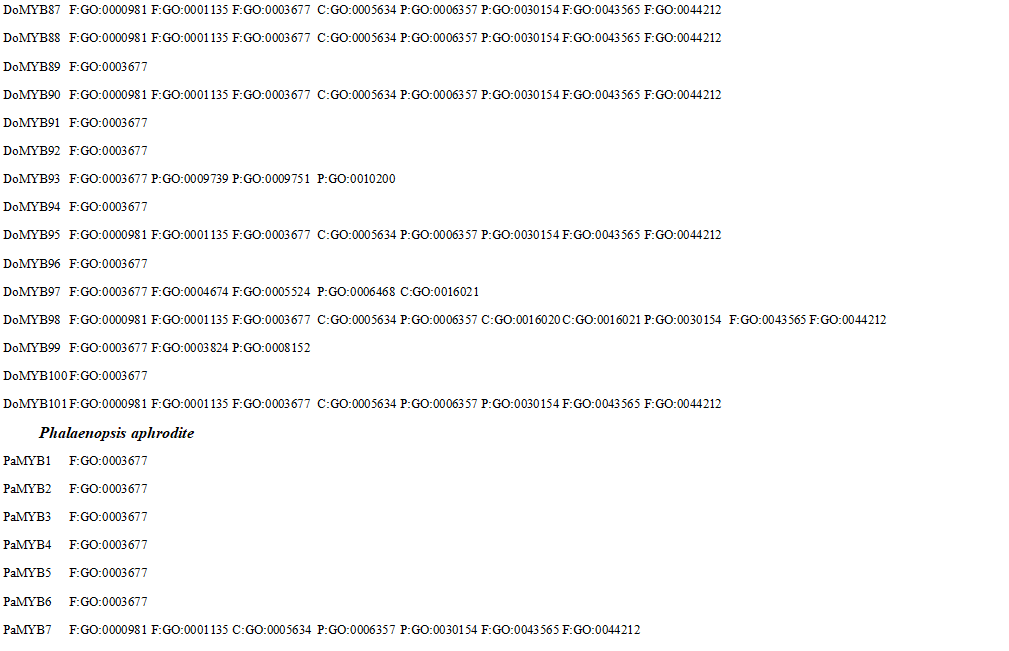


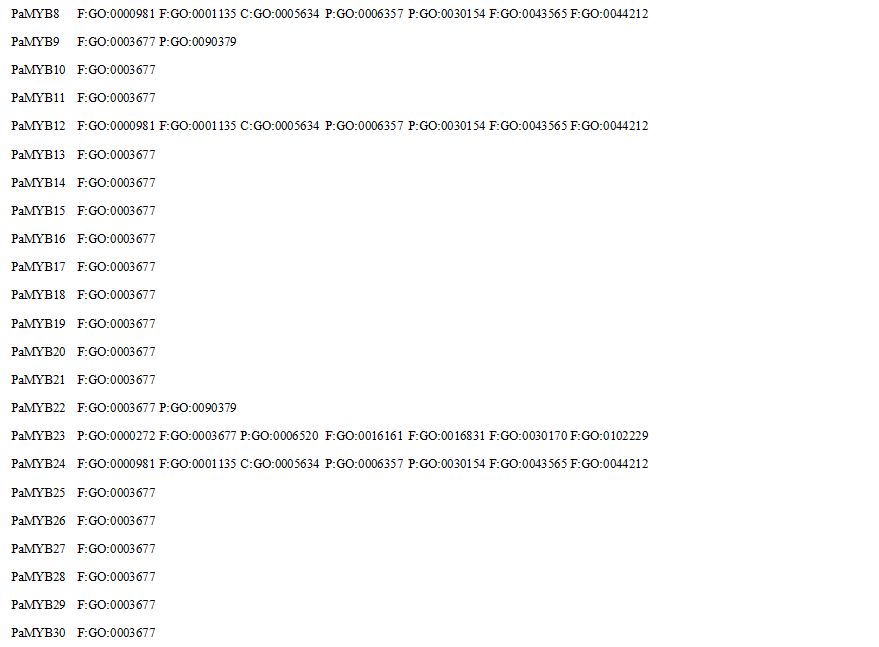


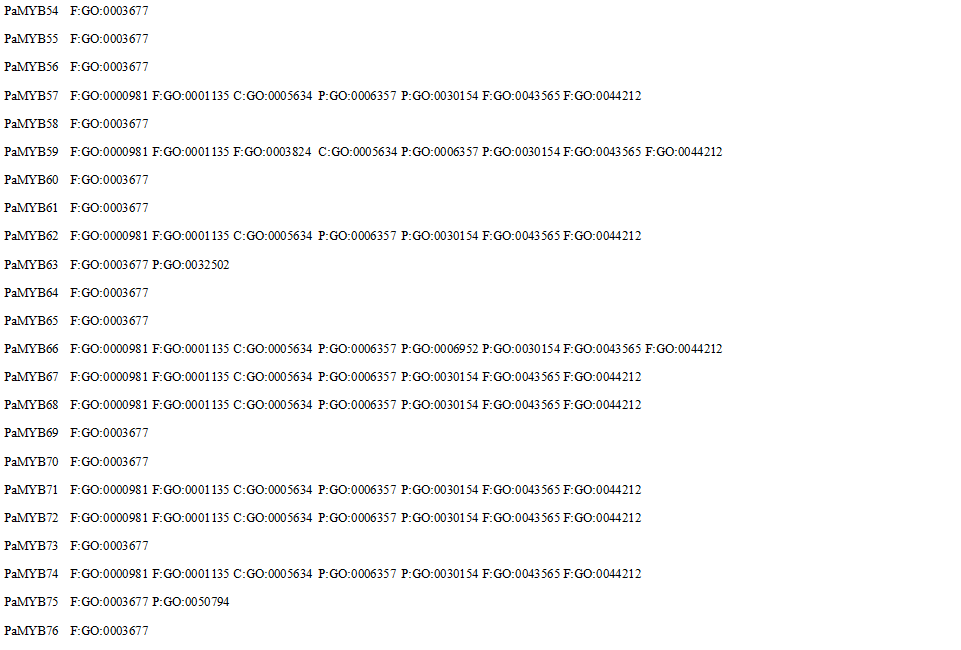

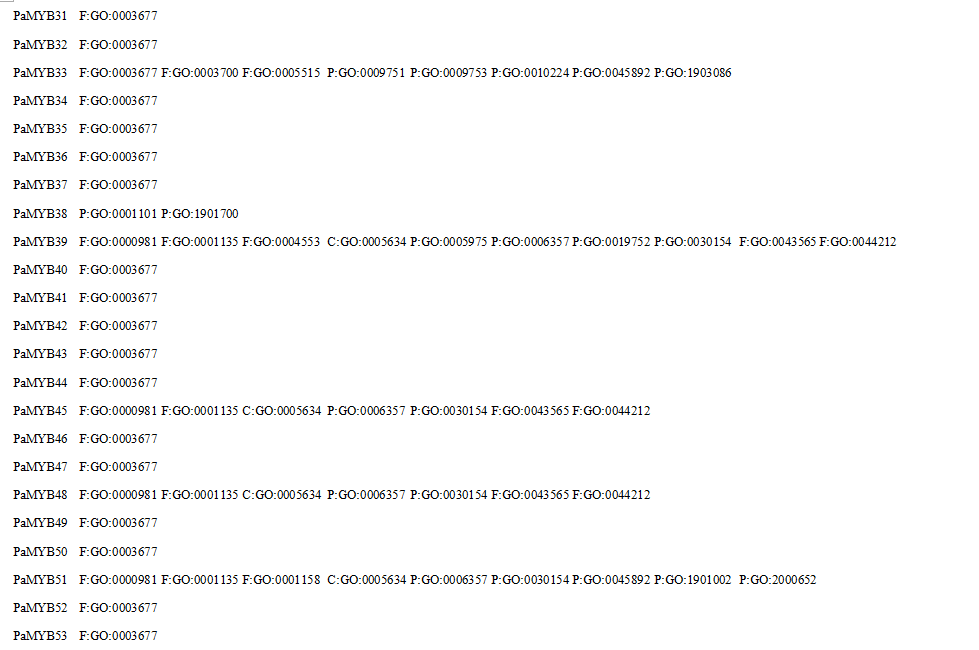


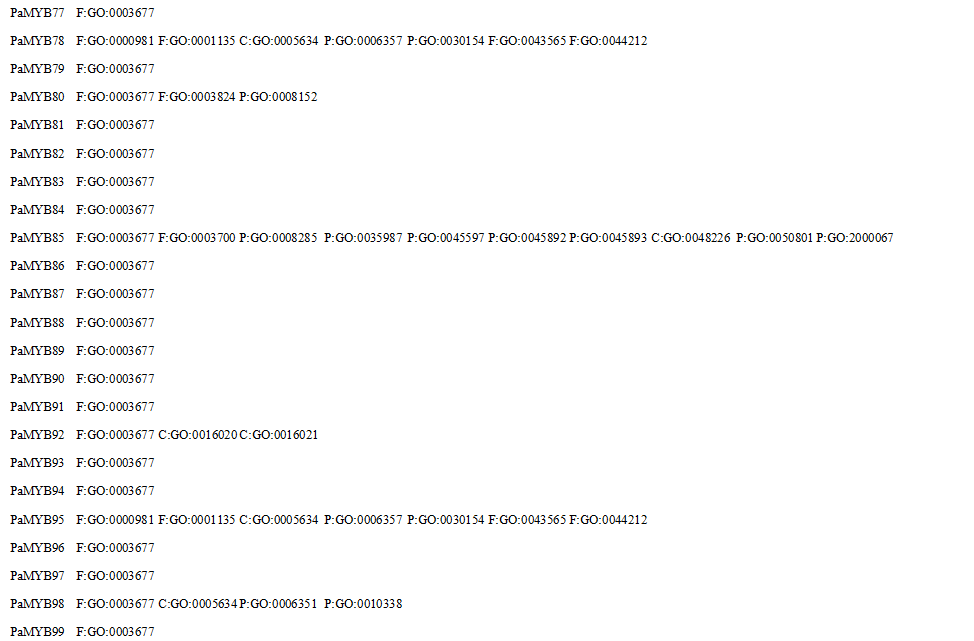


**Supplementary Table S6. The similarity values of R2R3-PaMYB cluster**

| **Gene 1** | **Gene 2** | **Similarity values** |
| --- | --- | --- |
| PaMYB4 | PaMYB5 | 58.05% |
| PaMYB6 | PaMYB7 | 47.93% |
| PaMYB8 | PaMYB9 | 70.19% |
| PaMYB10 | PaMYB18 | 60.15% |
| PaMYB11 | PaMYB13 | 73.75% |
| PaMYB16 | PaMYB20 | 59.17% |
| PaMYB19 | PaMYB21 | 67.97% |
| PaMYB23 | PaMYB39 | 54.39% |
| PaMYB25 | PaMYB27 | 41% |
| PaMYB29 | PaMYB31 | 55.12% |
| PaMYB32 | PaMYB34 | 49.76% |
| PaMYB37 | PaMYB52 | 52.17% |
| PaMYB40 | PaMYB43 | 35.51% |
| PaMYB44 | PaMYB60 | 35.74% |
| PaMYB45 | PaMYB57 | 43.69% |
| PaMYB48 | PaMYB50 | 68.97% |
| PaMYB49 | PaMYB51 | 47.43% |
| PaMYB53 | PaMYB56 | 58.16% |
| PaMYB54 | PaMYB58 | 62.78% |
| PaMYB55 | PaMYB61 | 42.34% |
| PaMYB62 | PaMYB72 | 64.68% |
| PaMYB64 | PaMYB65 | 100% |
| PaMYB67 | PaMYB69 | 47.54% |
| PaMYB73 | PaMYB91 | 87.25% |
| PaMYB74 | PaMYB79 | 54.88% |
| PaMYB75 | PaMYB85 | 50.46% |
| PaMYB76 | PaMYB94 | 37.62% |
| PaMYB78 | PaMYB87 | 51.20% |
| PaMYB80 | PaMYB97 | 18.34% |
| PaMYB82 | PaMYB88 | 46.46% |
| PaMYB83 | PaMYB90 | 48.88% |
| PaMYB84 | PaMYB86 | 53.33% |
| PaMYB89 | PaMYB93 | 37.22% |


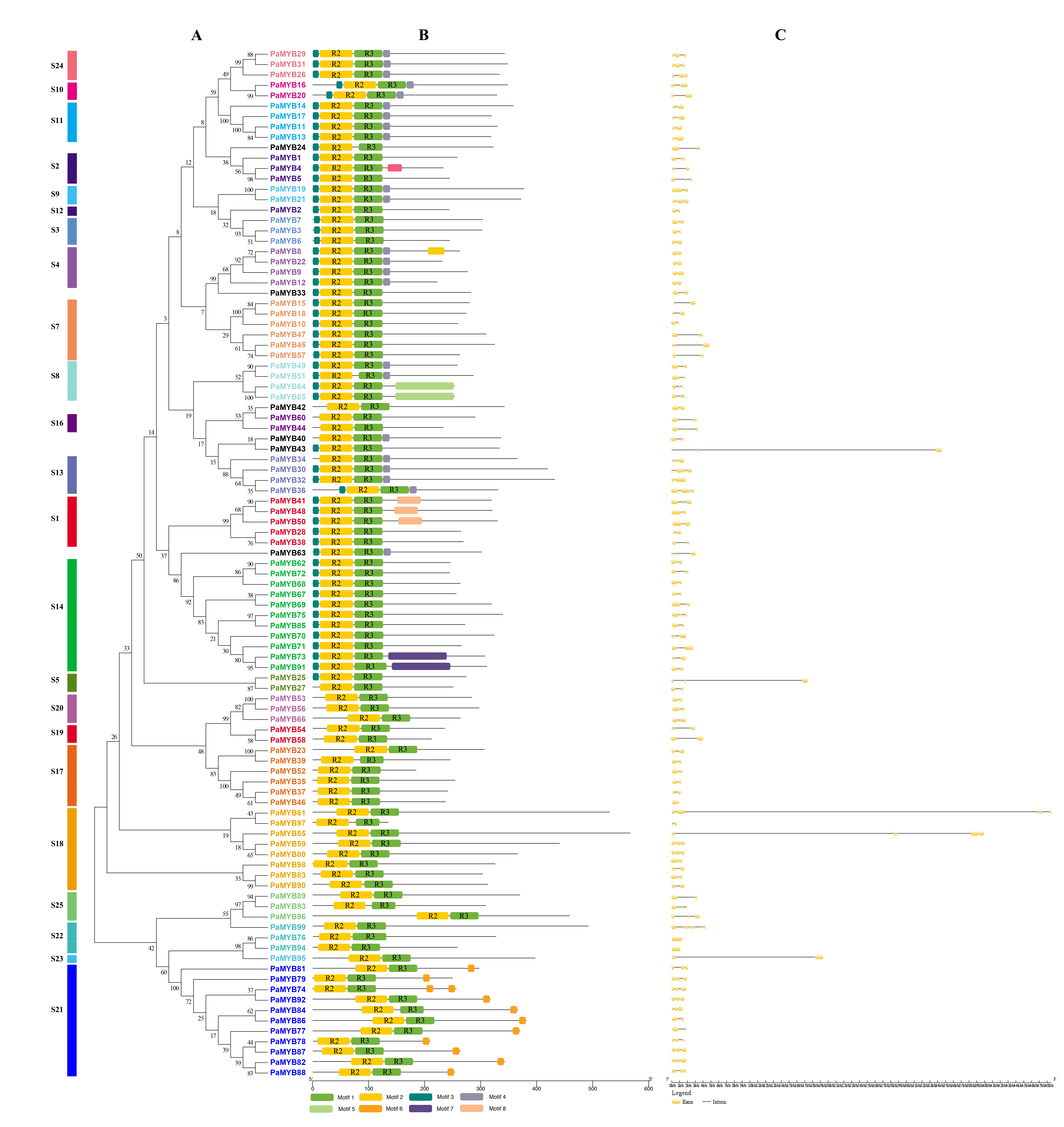


**Supplementary Fig. S1 Phylogenetic relationships, intron pattern, and architecture of conserved protein motifs in R2R3-MYB proteins from P. Aphrodite.** (B) The conserved motifs in the PaR2R3-MYB proteins were identified using MEME software. The grey lines represent nonconserved sequences, and each motif is indicated by a colored box numbered at the bottom. The lengths of the motifs in each protein are proportionally displayed; (C) Exon-intron structures were predicted and manually curated for the putative intact PaR2R3-MYB gene models. The yellow boxes and black lines represent exons and introns, respectively.


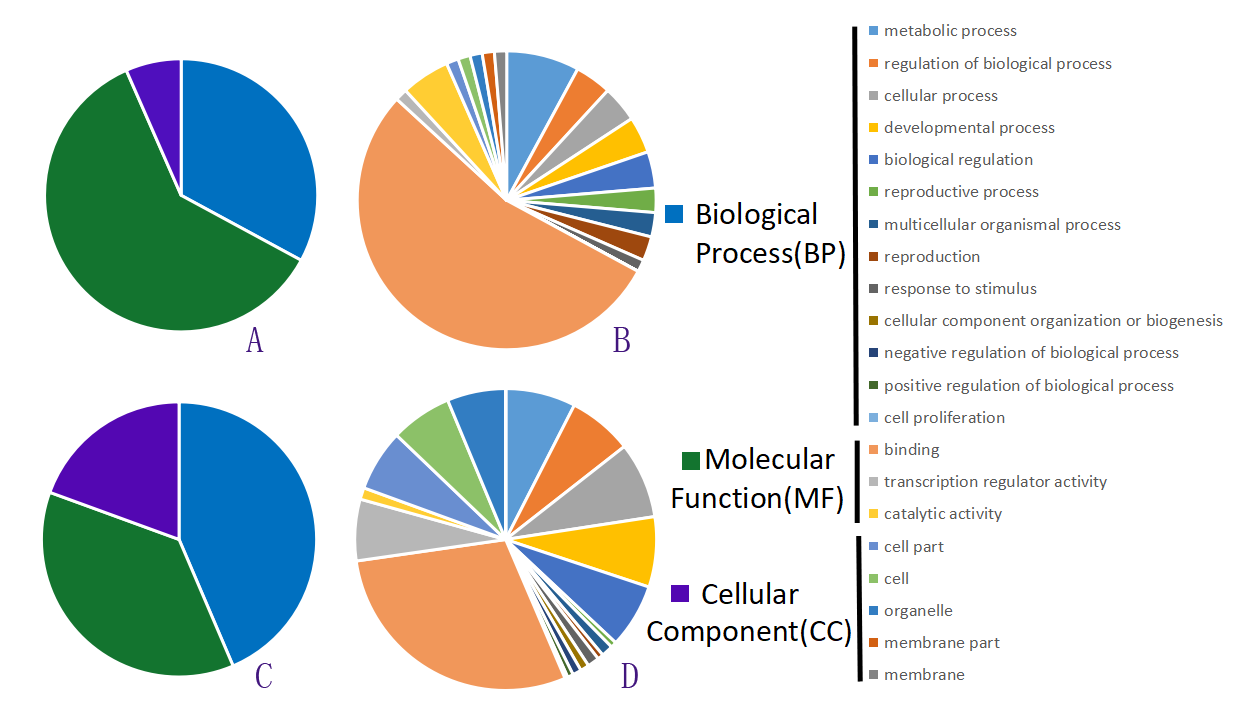


**Supplementary Fig. S2 Gene Ontology (GO) analysis of the R2R3-MYB genes from D. officinale (A/B) and P. aphrodite (C/D).** The size of each slice in the pie chart indicates the relative abundance of that GO term in the R2R3-MYBs.


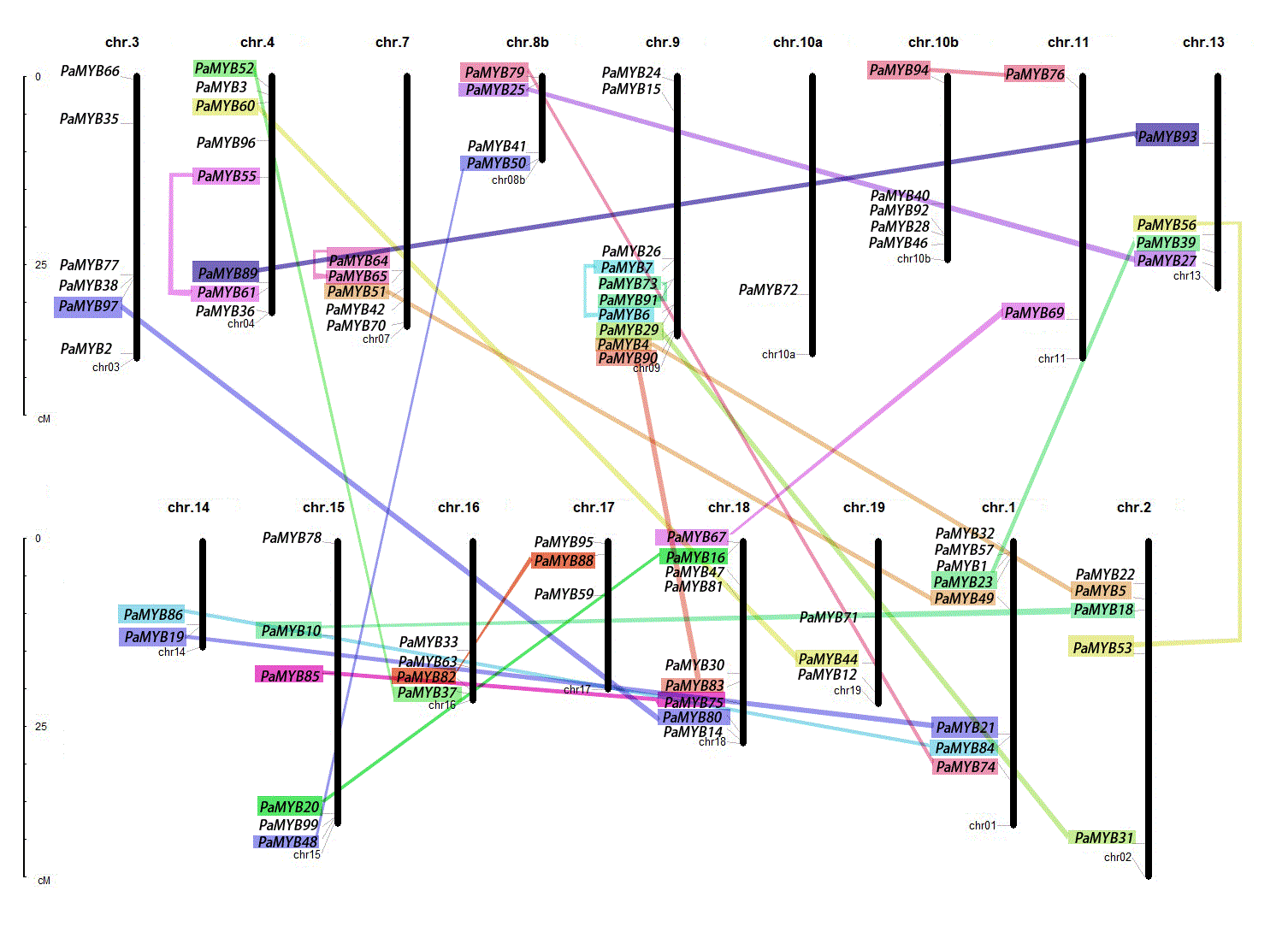


**Supplementary Fig. S3 Chromosomal location analysis of the PaMYB genes.** The colorful mark represented the PaMYB cluster. The chromosomal position of each PaMYB was mapped according to the GFF (General Feature Format) file from the online website (http://orchidstra2.abrc.sinica.edu.tw/orchidstra2/pagenome.php). The chromosome number is indicated at the end of each chromosome. The scale is in mega bases (Mb).


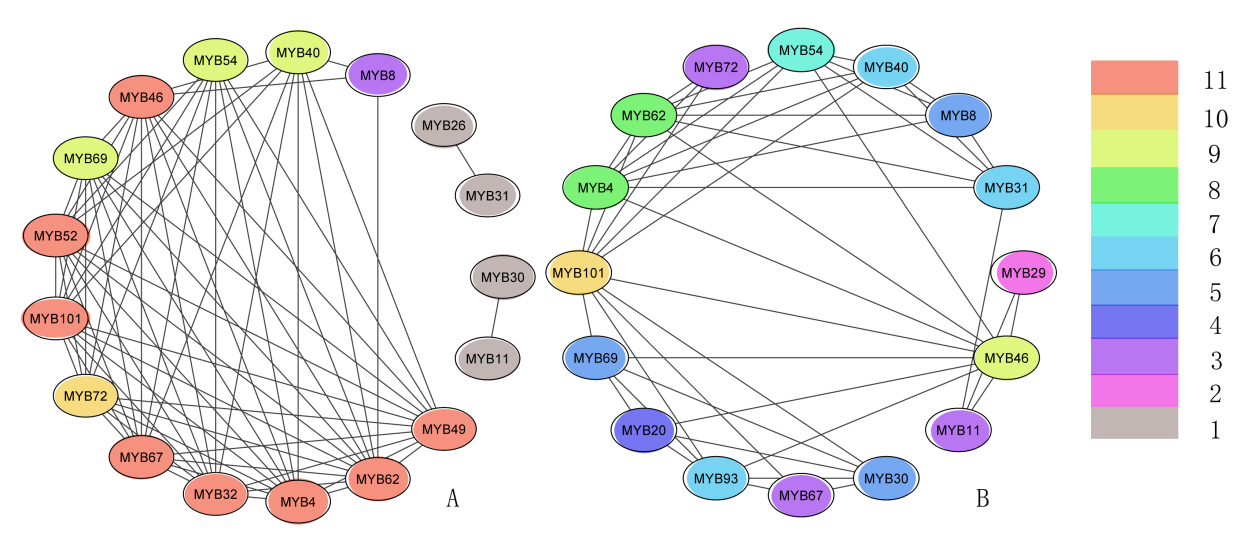


**Supplementary Fig. S4 Coexpression network of the *R2R3-DoMYB* genes in tissues (A) and their responses to salt, drought, MeJA, SA and ABA treatments (B).**  Relative expression of 20 *R2R3-MYB* genes under 300 mM NaCl, 300 mM mannitol and 100 μM abscisic acid (ABA) treatments at 0, 1, 2, 3, 4, 7 d. Relative expression of 20 R2R3-MYB genes under 100 μM methyl jasmonate (MeJA), 100 μM salicylic acid (SA) treatments at 0, 2, 4, 8, 24, 48 and 72 h.
